# Nearly 100% selective and visible-light-driven methane conversion to formaldehyde via. single-atom Cu and W^δ+^

**DOI:** 10.1038/s41467-023-38334-7

**Published:** 2023-05-10

**Authors:** Lei Luo, Xiaoyu Han, Keran Wang, Youxun Xu, Lunqiao Xiong, Jiani Ma, Zhengxiao Guo, Junwang Tang

**Affiliations:** 1 Key Lab of Synthetic and Natural Functional Molecule Chemistry of Ministry of Education, The Energy and Catalysis Hub, College of Chemistry and Materials Science, Northwest University, 710127 Xi’an, People’s Republic of China; 2 Department of Chemistry, The University of Manchester, Manchester, M13 9PL UK; 3 Department of Chemical Engineering, University College London, Torrington Place, London, WC1E 7JE UK; 4 Department of Chemistry, The University of Hong Kong, Pokfulam Road, 999077 Hong Kong, People’s Republic of China; 5 Present Address: State Key Laboratory of Catalysis, Dalian Institute of Chemical Physics, The Collaborative Innovation Centre of Chemistry for Energy Materials (iChEM), Dalian National Laboratory for Clean Energy, Chinese Academy of Sciences, Zhongshan Road 457, 116023 Dalian, People’s Republic of China

**Keywords:** Photocatalysis, Photocatalysis, Photocatalysis

## Abstract

Direct solar-driven methane (CH_4_) reforming is highly desirable but challenging, particularly to achieve a value-added product with high selectivity. Here, we identify a synergistic ensemble effect of atomically dispersed copper (Cu) species and partially reduced tungsten (W^δ+^), stabilised over an oxygen-vacancy-rich WO_3_, which enables exceptional photocatalytic CH_4_ conversion to formaldehyde (HCHO) under visible light, leading to nearly 100% selectivity, a very high yield of 4979.0 μmol·g^−1^ within 2 h, and the normalised mass activity of 8.5 × 10^6^ μmol·g^-1^_Cu_·h^−1^ of HCHO at ambient temperature. In-situ EPR and XPS analyses indicate that the Cu species serve as the electron acceptor, promoting the photo-induced electron transfer from the conduction band to O_2_, generating reactive •OOH radicals. In parallel, the adjacent W^δ+^ species act as the hole acceptor and the preferred adsorption and activation site of H_2_O to produce hydroxyl radicals (•OH), and thus activate CH_4_ to methyl radicals (•CH_3_). The synergy of the adjacent dual active sites boosts the overall efficiency and selectivity of the conversion process.

## Abstract (web summary)

Direct solar-driven methane (CH_4_) reforming is highly desirable but challenging. Here, the synergy of atomic Cu species and partially reduced tungsten (W^δ+^), stabilized over an oxygen-vacancy-rich WO_3_, enables exceptional CH_4_ conversion to formaldehyde (HCHO) under visible light.

## Introduction

The rapid transition towards net-zero carbon (CO_2_) emissions is an imperative undertaking by science and society to stave off potentially catastrophic climate change^1^. Hence, urgency arises: (1) to develop low-carbon transition pathways to turn traditional fossil resources into high-value-added chemicals, and (2) to displace traditional carbon-intensive manufacturing processes. Formaldehyde is an important high-volume industrial chemical with a market value of over 8 billion USD, expanding at a compound annual growth rate (CAGR) of 5.7%. It is widely used for household, commercial, aviation, medical and automotive products, and is a valuable precursor for melamine, urea-formaldehyde and phenolic resins, etc., due to its high reactivity and versatility^2^. It is also safely in use for the manufacture of vaccines, anti-infective drugs and hard-gel capsules. Currently, formaldehyde is produced by methanol oxidation-dehydrogenation using silver or metal-oxide catalysts at a high reactor temperature of over 500–600 °C, incurring both high CO_2_ emission and energy penalties.

On the other hand, with the continuous discovery of abundant methane (CH_4_) resources, especially shale/natural gas, the direct CH_4_ conversion into value-added chemicals such as methanol, formaldehyde and formic acid offers considerable economic and environmental benefits^3–7^. However, due to the high C–H bond dissociation energy (439 kJ·mol^−1^), CH_4_ serves as the most stable and inert industrial feedstock among alkanes^8–12^, and its industrial utilisation through indirect steam-reforming and subsequently Fischer-Tropsch synthesis is usually energy-intensive due to the high operating temperature (700–1100 °C)^13–16^. Therefore, sustainable CH_4_ utilisation under mild conditions is highly desirable.

As a renewable technology, photocatalysis has shown unprecedented opportunities for overcoming thermodynamic barriers and achieving direct CH_4_ conversion to various chemicals at ambient temperature. Recently, several efforts on direct photocatalytic CH_4_ oxidation to methanol (CH_3_OH) and formaldehyde (HCHO) have been reported. High selectivity to CH_3_OH (>90%) and HCHO (100%) but with very moderate yields (350 μmol·g^−1^·h^−1^ of CH_3_OH and 300 μmol·g^−1^·h^−1^ of HCHO), have been achieved over optimised FeO_*x*_/TiO_2_ and Au/WO_3_ photocatalysts, respectively^17,18^. Noble-metal (Au, Ag, Pd, Pt) modified ZnO and TiO_2_ performed acceptable yields of various oxygenates but with relatively low selectivity and mainly under UV/near UV irradiation (<80%)^17–23^. These advances encourage further investigations especially in the search for low-cost noble-metal-free photocatalysts with wide-spectrum response and simultaneous optimisation of activity and selectivity. Extending photoabsorption and enhancing charge separation generally improve photoactivity on various solar-driven reactions such as H_2_O splitting^24,25^, CO_2_ reduction^26,27^, organic synthesis^28,29^ and contaminant elimination^30,31^. However, such an approach is insufficient for CH_4_ conversion due to its rather low electron and proton affinity. Moreover, another challenge for the CH_4_ conversion into desirable oxygenates (i.e., CH_3_OH and HCHO) is that the targeted oxygenates are usually more reactive than CH_4_, which tend to be over-oxidised, leading to poor selectivity^23,32^. Hence, rationally regulating CH_4_ reaction dynamics and promoting charge separation are equally significant to tune the overall photocatalytic performance.

In industry, HCHO is solely produced from CH_3_OH oxidation and accounted for 30% consumption of CH_3_OH^33^. Multi-step production from CH_4_ via steam reforming, Fischer-Tropsch synthesis and methanol conversion is energy-intensive and requires harsh conditions^33,34^. Nevertheless, one-step conversion from CH_4_ to HCHO with high selectivity and yield is still lacking and no commercial catalyst is available even for high temperature. Hence, it is highly desirable to develop a suitable catalyst and/or co-catalyst as a promising alternative to promote photoabsorption, enhance charge separation and optimise selectivity under mild conditions^35^. The key to realise such direct conversion and selectivity depends on regulating the reactive oxygen species (ROS), enhancing the conversion of methane and promoting timely desorption of the desired products, whilst suppressing its mineralisation to CO_2_^18^. Suitable redox cocatalysts can be beneficial to charge transfer and promote the activation of the adsorbed O_2_ and H_2_O to form reactive oxygen species such as superoxide (·O_2_^-^), hydroperoxyl (·OOH) and hydroxyl (·OH) radicals. Introducing point defects in the photocatalyst may also modulate local electronic environment, facilitating reactant polarisation, chemical adsorption and hence electron/hole trapping to promote charge separation^36,37^. The integration of such dual reaction sites can simultaneously address the concerns on both charge separation and surface reaction dynamics^38,39^. Besides, a highly dispersed co-catalyst, especially single atoms or atomic clusters with a low co-ordination number, provides unique reaction sites for ready identification of the active local environment for CH_4_ conversion^40–42^. Accordingly, it is of considerable benefit to develop highly dispersed noble-metal-free dual-site coordinated catalysts.

Herein, tungsten oxide (WO_3_) nanocrystals were used as the substrate due to its visible-light responsive property largely extending natural light utilisation. Atomic copper co-catalyst and W^δ+^ sites associated with oxygen vacancies (O_v_) were hybridised to regulate synergistically charge transfer and surface reaction dynamics. Under 420 nm light irradiation, CH_4_ was converted to HCHO with 96.5% selectivity and a maximum time yield of 12.4 μmol·h^−1^. Computational simulations indicated that O_v_ are essential to stabilise the single Cu atoms and induce the formation of adjacent W^δ+^ sites. Further mechanistic investigations proved that Cu species acted as the electron acceptors, while W^δ+^ species facilitated hole transfer and the preferred adsorption and activation of H_2_O to generate reactive hydroxyl radicals and then activated CH_4_. The ensemble co-ordination of the adjacent dual sites of single Cu atoms and W^δ+^ species thus resulted in the superior activity and selectivity of CH_4_ conversion into HCHO at ambient temperature.

## Results and discussion

### Highly selective methane oxidation to formaldehyde by dioxygen

**Fig. 1:**
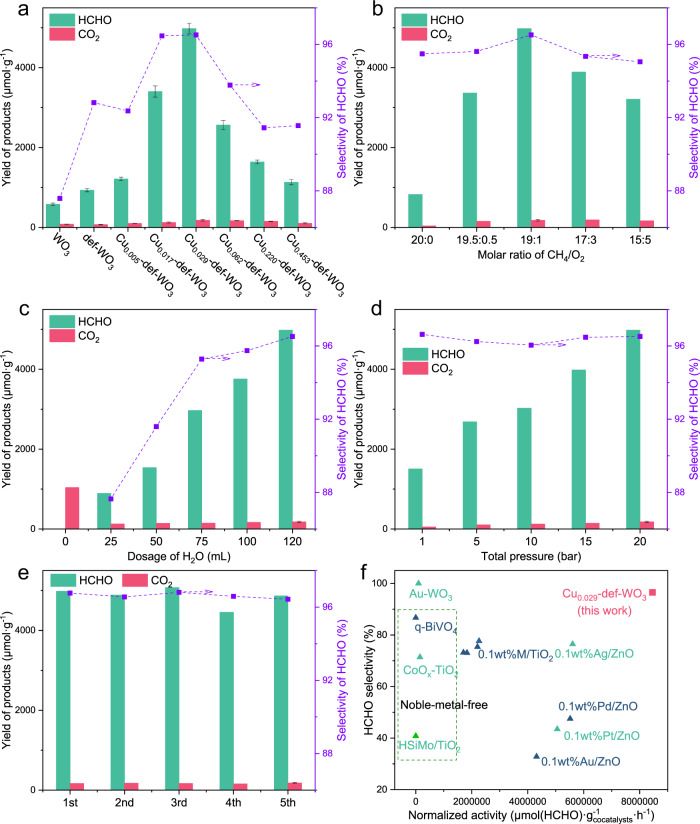
Selective CH_4_ oxidation. **a** HCHO production over WO_3_, def-WO_3_ and Cu_x_-def-WO_3_ photocatalysts in 2-h reaction. Optimisation of **b** molar ratio of CH_4_ to O_2_, **c** H_2_O dosage and **d** total pressure of CH_4_ and O_2_ mixture on HCHO production over Cu_0.029_-def-WO_3_. **e** Reusability of Cu_0.029_-def-WO_3_ under five-cycle run. **f** Comparison of CH_4_ conversion to HCHO over Cu_0.029_-def-WO_3_ and the typical reported photocatalysts^10,18–20,77^. Reaction conditions in **a**: 5 mg photocatalyst, 120 mL distilled H_2_O, 420 nm light irradiation and operated at 25 ^o^C. Reaction conditions in **b**, **c**, **d** are identical to **a** except varied O_2_ pressure, H_2_O dosage and total pressure, respectively.

CH_4_ conversion reaction was conducted under 420 nm light irradiation with 120 mL distilled H_2_O, 1 bar O_2_ and 19 bar CH_4_ for 2 h at 25 °C. With no photocatalyst or light irradiation, no HCHO or other oxygenate products was detected, suggesting the critical role of photocatalyst and light irradiation. Over pristine WO_3_, relatively low yields of HCHO (579.5 μmol·g^−1^) and CO_2_ (82.1 μmol·g^−1^) were produced (Fig. 1a), which was mainly ascribed to the severe charge recombination over single-component nanocrystals compared with multicomponent photocatalysts that could facilitate charge separation and transfer^24^. The selectivity of HCHO was calculated to be 87.6%. To enhance photocatalytic CH_4_ conversion efficiency, O_v_ linked with W^δ+^ sites, as point defects, were introduced, due to the potential advantages of (i) improving light absorption through injection of sub-band gap or traps^43^, (ii) enhancing charge separation and transfer^36^, (iii) promoting chemical adsorption and activation of the symmetric molecule^37^. Defective WO_3–*x*_ (denoted def-WO_3_) was prepared through temperature-programmed thermal reduction from pristine WO_3_ in a hydrogen atmosphere (5 vol.% H_2_/Ar). Compared with the pristine counterpart, def-WO_3_ showed 1.6 times higher HCHO production, being 933.2 μmol·g^−1^. Moreover, only a trace amount of CO_2_ by-products (72.2 μmol·g^−1^) was detected, indicating high selectivity of HCHO (92.8%) and significantly suppressed over-oxidation of CH_4_ to CO_2_ or CO.

To further promote HCHO production, all period 4 transition metal elements, including Sc, Ti to Cu and Zn, were hybridised with the defective WO_3–*x*_ photocatalyst via. a highly reproducible impregnation method, along with subsequent thermal reduction. The as-prepared photocatalysts with different metal contents were denoted M_*x*_-def-WO_3_, where *x*% represented the actual mass percentage of the specific metal (M). The actual metal content was measured by inductively coupled plasma optical emission spectrometry (ICP-OES). Reaction evaluation under identical conditions (Fig. S1) suggested that Cu was the most suitable co-catalyst for HCHO production among these 3d metals. The intrinsic reason why Cu is the most effective among the selected 3d candidates lie not only in its ability to catalyse oxygen reduction reaction, but also its synergistic cooperation with the substrate to promote H_2_O oxidation so as to activate CH_4_ as discussed below.

The effect of copper content on HCHO production was then optimised, which exhibited a volcanic trend with increasing percentage of Cu, as shown in Fig. 1a. The highest HCHO yield was over Cu_0.029_-def-WO_3_ (4979.0 μmol·g^−1^). Excessive Cu results in clustering, which may provide recombination centres for the charge carriers^44^. The dramatically enhanced activity is attributed to the increased availability of photo-generated charge carriers and efficient reactant activation as discussed below. Apart from the excellent HCHO production, a superior HCHO selectivity of 96.5% was also achieved. Moreover, a higher production rate means a higher HCHO concentration around the catalytic site, which may cause over-oxidation. However, no discernible level of CO_2_ was detected, suggesting that the produced HCHO molecules could be desorbed from the surface of the catalyst in time to avoid deep oxidation.

Effect of oxygen vacancy density on photocatalytic performance was also investigated by subtle control of the thermal reduction temperature with the same impregnated precursors. It was expected that a higher reduction temperature would lead to more O_v_ via. oxygen extraction by H_2_. As shown in Fig. S2, HCHO production reached the highest when it was prepared at 250 °C. Too high a temperature may result in the generation of deep trapping sites for charge recombination, while too low a temperature may not generate sufficient regulation of the structural defects.

As shown in Fig. 1b–d, the reaction conditions, including the molar ratio of CH_4_ to O_2_, H_2_O dosage and total pressure, were studied to optimise the HCHO production over Cu_0.029_-def-WO_3_. Initially, the effect of the molar ratio of CH_4_ to O_2_ was analysed, at a fixed pressure of 20 bar with varied CH_4_ and O_2_ dosages (Fig. 1b). Without O_2_ dosage, the HCHO conversion was 823.9 μmol·g^−1^ over Cu_0.029_-def-WO_3_, suggesting H_2_O could be the alternative oxygen source for HCHO. In the presence of a very small amount of molecular oxygen, HCHO production gradually increased with raising O_2_ pressure. Further increasing O_2_ while decreasing CH_4_ pressure led to a reduction of HCHO production to 3210.2 μmol·g^−1^ at CH_4_/O_2_ = 15/5. Such suppressed photoactivity would be primarily attributed to the reduced concentration of dissolved CH_4_. The optimal molar ratio of CH_4_ to O_2_ is 19:1, which is clearly higher than the stoichiometric ratio for the production of HCHO (CH_4_ + O_2_ → HCHO + H_2_O) and suggests a relatively large chemical gradient is needed to achieve an effective collision rate of the reactants at the catalytic local environment.

Secondly, H_2_O dosage was studied under the optimised CH_4_/O_2_ molar ratio (19/1). According to Raoult’s law, the molar ratio of the dissolved CH_4_/O_2_ is fixed in water. By variation of the H_2_O dosage, the concentration of the photocatalyst varies, further influencing the light transmittance. As shown in Fig. 1c, the higher the H_2_O dosage, the higher the HCHO production. The trend is almost linear: With the gradual increase of H_2_O content from 25 to 120 mL, the HCHO production is enhanced from 890.5 μmol·g^−1^ to 4979.0 μmol·g^−1^, corresponding to a concentration of HCHO from 178.1 μmol·L^−1^ to 207.5 μmol·L^−1^, respectively (Fig. 1c). The amount of oxidation of HCHO to CO_2_ would be higher at a higher concentration of HCHO, as indeed observed here. The CO_2_ production increases from 125.5 to 166.2 μmol·g^−1^ when water dosage is changed from 25 to 120 mL, or the concentration of HCHO varies from 178.1 to 207.5 μmol·L^−1^. Interestingly, an improved selectivity to HCHO from 87.6% to 96.5% is also observed, suggesting that the change in CO_2_ production does not affect the selectivity. Moreover, the HCHO production increases by ~4.6 folds from 890.5 to 4979.0 μmol·g^−1^ for the H_2_O dosage increase from 25 to 120 mL, respectively. Therefore, the dilution (or more water content) should preferentially promote the production and timely desorption of HCHO, rather than its oxidation on the surface of the photocatalyst. In other words, the increased HCHO production and selectivity can be attributed to the boosted light absorption and mass transfer of the products as a result of the higher amount of water used, at least under the range of H_2_O dosages considered here. More importantly, without H_2_O, a larger amount of CO_2_ (1038.8 μmol·g^−1^) is detected, suggesting that H_2_O can suppress HCHO over-oxidation into CO_2_. This may be attributed to the solvation effect of H_2_O, promoting the desorption of oxygenate products^23,45^.

Thirdly, the HCHO production was assessed over varying feeding pressure of CH_4_ and O_2_ under a constant molar ratio of 19 (Fig. 1d). It reveals that HCHO production over Cu_0.029_-def-WO_3_ responded almost linearly to the total feeding pressure. As mentioned above, this indicates that no side reaction occurred when increasing the dissolved CH_4_/O_2_ at the constant ratio. The intrinsic HCHO product reached 1505.7 μmol·g^−1^ at ambient pressure. Finally, as shown in Fig. 1e, Cu_0.029_-def-WO_3_ performed a relatively stable CH_4_ conversion to HCHO, with no evident deactivation after five cycles, which confirms the stability of Cu_0.029_-def-WO_3_ as a desirable photocatalyst. Meanwhile, the XRD (Fig. S3) and XPS spectra (Fig. S4) comparison of the freshly prepared Cu_0.029_-def-WO_3_ and the one after 5 cycles exhibited no discernible difference. Meanwhile, copper was not detected in the filtered reactant by ICP-OES, which further suggests the stable topology of Cu_0.029_-def-WO_3_.

The normalised mass activity was calculated using the formula (${{{{{\rm{Normalized\; activity}}}}}}=\,\frac{{{{{{\rm{n}}}}}}}{{{{{{\rm{m}}}}}}*{{{{{\rm{t}}}}}}}$), where *n*, *m* and *t* represent the molar production of HCHO (μmol), the mass of Cu co-catalyst (g) and reaction time (h), respectively. Comparison between the Cu_0.029_-def-WO_3_ photocatalyst and typically reported photocatalysts for CH_4_ conversion to HCHO is shown in Fig. 1f. Most of the photocatalysts show relatively low normalized activity for HCHO production. Some noble-metal-modified ZnO and TiO_2_ photocatalysts under full-arc irradiation exhibit a higher normalized activity between 1.8 × 10^6^ to 5.5 × 10^6^ μmol_(HCHO)_·g^−1^_(cocatalyst)_·h^−1^, but the selectivity for HCHO is lower than 80%^19^. Regarding the selectivity, the best-performed Au-WO_3_ catalyst presents a 100% HCHO selectivity but more than serval order of magnitudes lower normalized activity than our work^18^. Comparatively, the present noble-metal-free Cu_0.029_-def-WO_3_ photocatalyst shows much superior photocatalytic activity with nearly 100% HCHO selectivity and a normalized activity as high as 8.5 × 10^6^ μmol_(HCHO)_·g^−1^_(cocatalyst)_·h^−1^ (with an apparent quantum yield of 1.14%) under 420 nm irradiation.

### Observation of active sites

**Fig. 2:**
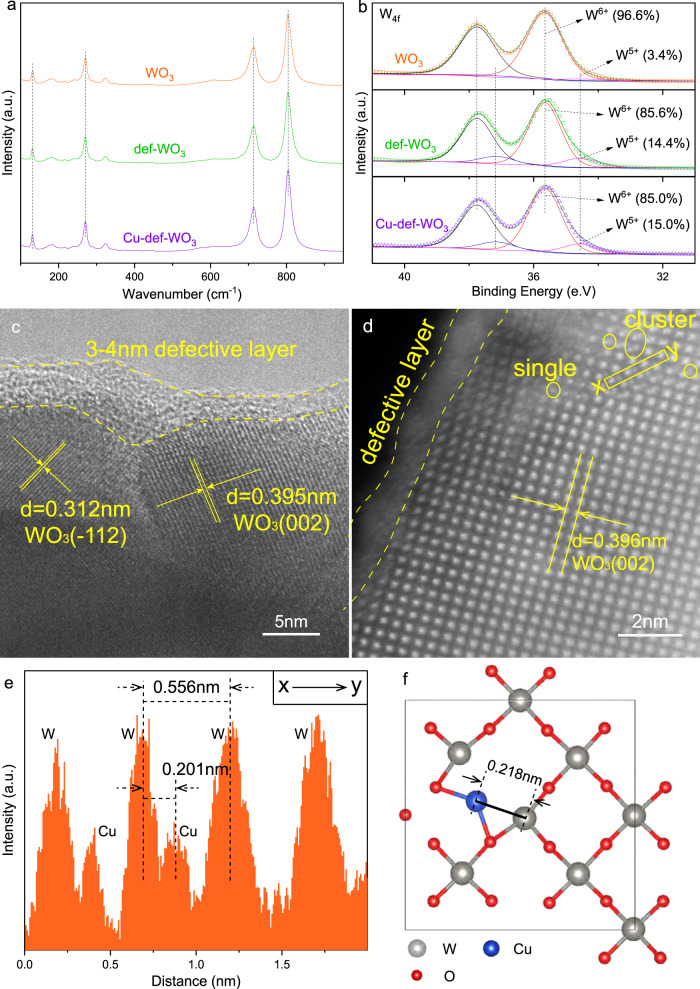
Structural characterisation. **a** Raman spectra and **b** high-resolution W_*4f*_ XPS spectra of WO_3_, def-WO_3_ and Cu_0.029_-def-WO_3_. **c** HRTEM and **d** aberration corrected HAADF-STEM images of Cu_0.029_-def-WO_3_. **e** *x*–*y* line scan curve measured along the yellow rectangle region marked in **d**. **f** The optimised configuration of the atomic Cu at A site of def-WO_3_ over the (002) surface in the top view.

In order to clarify the mechanism of the high performance, we compared three samples (WO_3_, def-WO_3_ and Cu_0.029_-def-WO_3_), all of which show a typical and well-crystallised monoclinic WO_3_ crystal structure (PDF#43-1035) as noted in the X-ray diffraction patterns (XRD) (Fig. S5). The incorporation of copper species through high-temperature reduction did not change the XRD patterns, indicating the stable topology of the WO_3_ nanocrystal. In addition, there are no extra copper-associated diffraction peaks, due to its high dispersion and/or low loading. Raman spectra (Fig. 2a) further support the WO_3_ crystalline structure according to the typical WO_3_ peaks observed at 130.8, 270.4, 713.2 and 804.2 cm^−1^, which are contributed by lattice vibration, δ(O-W-O) deformation vibration and stretching ν(O-W-O) mode of the bridging oxygen of WO_3_, respectively^46^.

High-resolution X-ray Photoelectron Spectroscopy (XPS) was conducted to examine the chemical structure of the defective surface of the photocatalyst. As shown in Fig. 2b, the two deconvolution peaks from the W_*4f*_ XPS spectra at 35.65 and 34.56 eV are observed for the pristine WO_3_ and attributed to W^6+^ and W^5+^ species, respectively. The minor amount of W^5+^ species (3.4%) could be ascribed to the low concentration of the intrinsic defects in the nanocrystal^47^. For the def-WO_3_, the level of the W^5+^ species increased to 14.4%, over 4 times higher than that of the counterpart, suggesting the successful introduction of O_v_ linked with the W^5+^ species. High-resolution O_1s_ XPS spectra (Fig. S6) provides further evidence of the successful introduction of O_v_, where the peak at 531.77 eV can be deconvoluted and ascribed to the O_v_^48^. The other two peaks centred at 530.32 and 532.88 eV can be assigned to the crystalline oxygen (W-O) and adsorbed moisture, respectively^49^. Accordingly, the results indicate that the hydrogen reduction is effective to introduce surface O_v_ species, which regulate the local electronic environment and generate W^δ+^ sites therein^50–52^. Similar to those in the def-WO_3_, the percentages of W^5+^ and O_v_ species of the Cu_0.029_-def-WO_3_ are 15.0% and 8.83%, respectively. This indicates that the O_v_ are preserved after the Cu loading. Besides XPS spectra, O_v_ are also confirmed through TEM images, EPR and UV-DRS as discusses later.

**Fig. 3:**
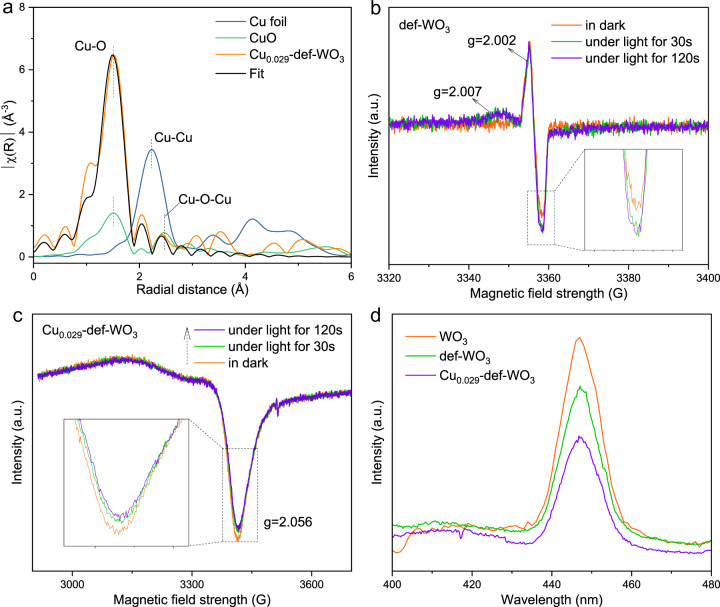
Structural identification and photo-physical properties. **a** Fourier transforms of EXAFS of the Cu K-edge of Cu_0.029_-def-WO_3_, CuO and Cu-foil. Low-temperature in situ solid-state EPR spectra of **b** def-WO_3_ and **c** Cu_0.029_-def-WO_3_ under 420 nm irradiation for different time. **d** Steady-state PL spectra of WO_3_, def-WO_3_ and Cu_0.029_-def-WO_3_.

The transmission electron microscopy (TEM) images of the pristine WO_3_ and the def-WO_3_ are shown in Fig. S7a, b and S8, respectively. The former is characterised by a well-crystallised nanocrystal structure with smooth surfaces. High-resolution transmission electron microscopy (HRTEM) is accordance with the XPS analysis. Such defects in the subsurface are reported to result in electron delocalisation and benefit the stabilisation of reaction intermediates^53–55^. The crystalline d-spacings of 0.395 and 0.312 nm in Fig. 2c can be ascribed to (002) and (−112) facets of the WO_3_ nanocrystal, respectively, indicating the primary structure of WO_3_ is retained, consistent with the XRD observation. The unsaturated sites of the amorphous/defective layer should promote chemical adsorption and activation of the reactants^43^. Aberration corrected high-angle annular dark field scanning transmission electron microscopy (HAADF-STEM) images (Fig. 2d) were captured to further investigate the dispersion of the copper species. Irregular tungsten atoms that present on the edge of the nanocrystal further support the existence of O_v_-induced W^δ+^ sites on the Cu_0.029_-def-WO_3_. Besides, it is also shown that W atoms distributed in the regular array, while some additional and smaller dots are clearly observed among the regular array, which may be assigned to Cu atoms. Cu K-edge X-ray absorption near edge structure (XANES) spectra of Cu_0.029_-def-WO_3_ with Cu-foil and CuO references were then measured to further study the structural microenvironment of the Cu atoms (Fig. S10). Fourier transforms of the Cu K-edge (Fig. 3a) exhibit only a predominant peak at ca. 1.51 Å for Cu_0.029_-def-WO_3_, which can be ascribed to the first shell of the Cu–O bond with reference to the CuO sample (Fig. S11 and Table S1). In parallel, no peaks corresponding to Cu-O-Cu and Cu-Cu at 2.47 Å and 2.23 Å were detected, confirming that the atomically dispersed Cu sites exist in the Cu_0.029_-def-WO_3_ and are coordinated with oxygen.

The *x*–*y* elemental line scan (Fig. 2e) along with the yellow rectangle of Fig. 2d clearly shows the atomic dispersion of Cu in the oxide lattice. The distance between adjacent W and Cu atoms was measured as 0.201 nm along [001] crystalline direction of the (002) plane, which is consistent with the following density functional theory (DFT) calculation results that the O_v_ is energetically favourable to host a single atomic Cu dopant (Fig. 2f and Fig. S12), where the direct line distance between the nearest W and Cu atoms is 0.434 nm. When projected normal to the [001] direction along the (002) plane, it is 0.218 nm, close to the experimental observation. Thus, the pristine (002) surface model was built based on the monoclinic WO_3_ bulk structure, shown in Fig. S13a, b. The bottom two layers were fixed, while all other atoms were fully relaxed until the energy and force criteria were reached. The distance of the (002) surface is 3.91 Å, close to the experimentally measured value in this study (3.95 Å). The oxygen vacancy was created by the removal of a single oxygen atom on the top surface. After relaxation, the distance of the nearest W atom stretched to 4.34 Å (Fig. S14a), compared to 3.85 Å in the pristine case. This open-site gives the energetically most favourable host position for an atomic Cu. Three possible adsorption sites were comparatively investigated, as shown in Fig. S14b–d, denoted as Site A, B and C, respectively. The optimised configurations are shown in Fig. 2f. The adsorption energy was calculated based on:

1 $${E}_{{{{{{{\mathrm{ad}}}}}}}}={E}_{{{{{{{\mathrm{total}}}}}}}}-{E}_{{O}_{v}}-{E}_{{{{{{{\mathrm{Cu}}}}}}}}$$

where the ${E}_{{{{{{{\mathrm{total}}}}}}}}$, ${E}_{{O}_{v}}$ and ${E}_{{{{{{{\mathrm{Cu}}}}}}}}$ are the energy of the whole system, that of the system with a single O vacancy on the WO_3_ (002) surface and the chemical energy of copper, respectively. The results are listed in Table S2. Both the A and the B sites are energetically favoured, but the A site is the most stable. The Bader charge analysis was carried out to evaluate the charge changes, and the results are listed in Table S3. For the def-WO_3_, the Bader partial potential of the W atom decreases from −2.59e for the 2nd nearest to −2.34e for the nearest, suggesting a lowering of the valence of the nearest W^δ+^ (*δ* < 6), induced by the oxygen vacancy. Such reduced oxidation states of W around the defect were also the case for the Cu-def-WO_3_, which strongly proves the existence of the W^δ+^ species at the active site. By accommodating the Cu in the A site (the most stable structure, also consistent with the HAADF-STEM images), the oxidation states of W decreased by 13% from −2.52e to −2.19e. This lends further benefit to W and Cu as the dual active sites for water and oxygen adsorption, respectively, as discussed later.

### Mechanistic investigation

UV–Vis diffraction spectra (UV-DRS) (Fig. S15) show similar photoabsorption characteristics among WO_3_, def-WO_3_ and Cu_0.029_-def-WO_3_, suggesting that photoabsorption is not the dominant factor influencing the photocatalysis herein. The absorption boundary at 426 nm determines the visible-light responsive properties of the as-prepared photocatalysts. Bandgap energies calculated by the Tauc plots (Fig. S16) are 2.91, 2.88 and 2.88 eV for WO_3_, def-WO_3_ and Cu_0.029_-def-WO_3_, respectively. Additionally, Mott-Schottky plots (Fig. S17) were measured to establish the flat band position. Positive slopes of the Cu_0.029_-def-WO_3_ photocatalyst indicate the n-type characteristics of WO_3_, for which the flat band always lies 0.1 V below the conduction band (CB)^56^. Thus, CB and valence band (VB) positions of the Cu_0.029_-def-WO_3_ vs. NHE (pH = 0) are established as −0.10 and 2.78 V, respectively^57,58^. Therefore, the potentials of VB and CB positions are sufficient to drive O_2_ reduction (−0.05 V vs. NHE) and H_2_O oxidation (2.38 V vs. NHE) to generate reactive species, such as ·OOH and ·OH radicals, respectively^59,60^.

Electron paramagnetic resonance (EPR) is a highly sensitive tool for the study of the paramagnetic transition of metal ions and the oxygen vacancies due to unbalanced electron spins^61^. As shown in Fig. S18, no evident EPR signal was detected for WO_3_, suggesting its pristine topology with no unpaired electrons. After the thermal reduction in hydrogen, the def-WO_3_ exhibited a single Lorentzian EPR signal at *g* = 2.002, which can be assigned to an oxygen defective structure^49^. In the case of the Cu_0.029_-def-WO_3_, a similar EPR signal at *g* = 2.002 was also observed, but weaker than that of the def-WO_3_, thus indicating that the introduced copper species are very likely on/around O_v_ so reducing its EPR response^62^. Meanwhile, an additional hyperfine peak at *g* = 2.061 is observed, which is attributed to the hybridized Cu^2+^ species^63,64^. The hyperfine structure of this peak provides further evidence of the high dispersion of the copper species.

In situ light-irradiated EPR spectra of the def-WO_3_ and the Cu_0.029_-def-WO_3_ were then tested to probe further details of the charge transfer mechanism. For the def-WO_3_, a newly added EPR signal at *g* = 2.007 was observed under light irradiation (Fig. 3b), which is likely due to the excited electrons at the conduction band of WO_3_. In parallel, the signal of O_v_ at *g* = 2.002 gradually becomes stronger under light irradiation, suggesting O_v_ facilitate the trapping of active electrons from the conduction band and an equilibrium is reached after 120 s. In contrast, no noticeable change at *g* = 2.007 and 2.002 (Fig. S19) was observed for the Cu_0.029_-def-WO_3_ under identical conditions, suggesting that the photo-induced electrons at the conduction band of WO_3_ and the O_v_ could efficiently migrate to the Cu species. In addition, the constant intensity of the Lorentzian signal at *g* = 2.002 would suggest that the O_v_ serve as the mediator for electron transfer from the conduction band to the Cu species in the Cu_0.029_-def-WO_3_. Accordingly, a hyperfine EPR signal at *g* = 2.056 (Fig. 3c) was observed for the Cu_0.029_-def-WO_3_, which is ascribed to the Cu^2+^ species as Cu^+^ and Cu^0^ are EPR-silent. Under continued light irradiation, the Cu^2+^ signal gradually weakens, indicating a reduced content of the Cu^2+^ species, implying that the Cu^2+^ serves as the electron acceptor. In situ Cu_*2p*_ XPS spectra (Fig. S20) with and without light irradiation were obtained for further investigation of the role of the Cu species. In dark, the deconvoluted Cu_*2p*_ XPS results validate the coexistence of Cu^0^/Cu^+^ (932.38 eV) and Cu^2+^ (933.60 eV) for the Cu_0.029_-def-WO_3_, which corresponds to the composition of 66% Cu^0^/Cu^+^ and 34% Cu^2+^, respectively. Under light irradiation, the concentration of Cu^2+^ dramatically decreased to 19% while Cu^0^/Cu^+^ increased to 81%, further confirming the active role of Cu^2+^ as the electron acceptor.

**Fig. 4:**
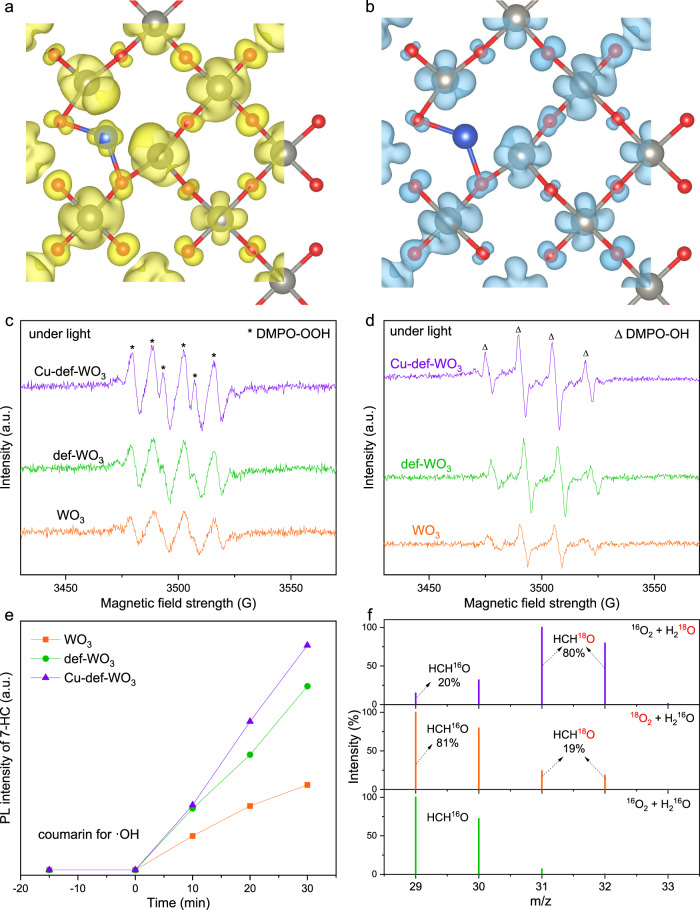
Photo-chemical properties. **a** Conduction band minimum and **b** Valence band maximum charge distributions of Cu-def-WO_3_. The grey, red and blue balls represent W, O and Cu atoms, respectively. In situ EPR monitor of **c** ·OOH and **d** ·OH radicals trapped by DMPO over WO_3_, def-WO_3_ and Cu_0.029_-def-WO_3_ under light. **e** Time-dependent PL intensity of 7-hydroxycoumain from the reaction of coumarin and ·OH over WO_3_, def-WO_3_ and Cu_0.029_-def-WO_3_. **f** GC-MS of the produced HCHO with isotopic labelled H_2_^18^O or ^18^O_2_ in the presence of 19 bar CH_4_ and 1 bar O_2_ and 3 mL H_2_O over Cu_0.029_-def-WO_3_.

Steady-state and time-decay photoluminescence (PL) spectra (Fig. 3d and S21) were conducted to study the charge transfer dynamics. The strong PL peak of the pristine WO_3_ is correlated with its severe charge recombination, as expected for the single component^24^. Comparatively, the def-WO_3_ shows relatively weaker PL intensity than WO_3_, indicating an enhanced charge separation efficiency^65^, which should originate from the role of O_v_ in the def-WO_3_ as the electron trapping sites. In the case of the Cu_0.029_-def-WO_3_, PL intensity decreased further, being the lowest, among the different Cu-based photocatalysts, implying the most suppressed charge recombination case after the incorporation of the atomic Cu. Notably, different from the def-WO_3_, the O_v_ in the Cu_0.029_-def-WO_3_ contribute little to charge separation, as evidenced by the unchanged EPR intensity under light irradiation (Fig. S19), suggesting the highly dispersed Cu co-catalyst is more efficient than the O_v_ in acting as the photo-induced electron acceptors. DFT simulations were then employed to show the charge density distribution of the conduction band minimum (Fig. 4a) and the valence band maximum (Fig. 4b) of the Cu-def-WO_3_. The charges are found to significantly accumulate around the Cu and the O_v_ region in the conduction band minimum (Fig. 4a). From Bader charge analysis in Table S3, the partial charge of the related O atom changes from 0.58e for the pristine WO_3_, to 1.06e for the def-WO_3_, and 0.98e for Cu-def-WO_3_. It confirms our speculations in EPR results. From the in situ EPR and XPS analyses, with further support from DFT simulations, it may be inferred that photo-excited electrons in the conduction band are firstly trapped by the O_v_, and then transferred to the Cu species to facilitate the formation of Cu^+^ (Cu^2+^ + e^-^ → Cu^+^), where Cu^+^ would next act as the electron donor to activate the adsorbed O_2_ on the surface (Cu^+^ + O_2_ → Cu^2+^ + ·OO^-^). Under reaction conditions in the presence of H_2_O, superoxide radicals (·OO^-^) tend to react with a proton and form ·OOH radicals. In parallel, photo-induced holes locate around the hybridized O and W^δ+^ (Fig. 4b).

Time-decay PL spectra (Fig. S22) were fitted by the two-exponential decay. All samples exhibit similar exponential emission properties. Compared with the WO_3_ and the def-WO_3_, a much slower decayed emission of the Cu_0.029_-def-WO_3_ demonstrates slower kinetics of the fluorescent decay and the suppressed charge recombination. The average PL lifetime of the Cu_0.029_-def-WO_3_ was calculated to be 4.61 ns (Table S4), longer than that of the WO_3_ (3.14 ns) and the def-WO_3_ (3.27 ns), indicating that the Cu_0.029_-def-WO_3_ most facilitates the charge carrier separation. Photocurrent responses were measured to evaluate further the charge separation dynamics. As shown in Fig. S23, the def-WO_3_ shows a photocurrent intensity of 12.2 μA·cm^−2^, 1.9 times stronger than that of the pristine WO_3_ (6.3 μA·cm^−2^), demonstrating the efficient transfer of photo-induced electrons to the defects. For Cu_0.029_-def-WO_3_, photocurrent density is further increased to 14.8 μA·cm^−2^, about 1.2 times higher than that of the def-WO_3_, suggesting the role of the Cu species in promoting charge separation. Electrochemical impedance spectroscopy (EIS) plots (Fig. S24) show a much smaller radius of Cu_0.029_-def-WO_3_ than those of def-WO_3_ and WO_3_, indicating its smallest resistance for interfacial charge transfer. From the above analysis, it is clear that the charge recombination is greatly suppressed by suitable ensemble corporation of the O_v_-W^δ+^ sites and highly dispersed Cu-co-catalyst atoms, which result in efficient charge separation and transfer.

Reactive oxygen species, including hydroperoxyl (·OOH) and hydroxyl radicals (·OH), were monitored with 5, 5-dimethyl-1-pyrroline N-oxide (DMPO) as the trapping agent by the detection of DMPO-OOH and DMPO-OH adducts, respectively. The intermediate of O_2_ reduction was measured in methanol solution under light irradiation (Fig. 4c). There were clearly six prominent characteristic signals of the DMPO-OOH adduct at hyperfine splitting constants of *A*_N_ = 15.4 G and *A*_H_ = 10.6 G over WO_3_, def-WO_3_ and Cu_0.029_-def-WO_3_ photocatalysts^66^. Cu_0.029_-def-WO_3_ and def-WO_3_ show 2.0 and 1.8 times stronger ·OOH production than the pristine WO_3_, due to the highly suppressed charge recombination induced by the cooperation of Cu single atoms and Ov. Figure 4d shows the four signal peaks (1: 2: 2: 1) of ·OH over the three photocatalysts. The trend of ·OH production from H_2_O oxidation with photo-induced holes (H_2_O + h^+^ → ·OH + H^+^) is similar to ·OOH production among WO_3_, def-WO_3_ and Cu_0.029_-def-WO_3_, due to the enhanced charge separation. Quantification experiments further support that the production of ·OH radicals is directly associated with charge separation. The ·OH test using coumarin as the probe molecule (Fig. 4e) supports that the Cu_0.029_-def-WO_3_ is more efficient than the WO_3_ and the def-WO_3_. Compared with the pristine WO_3_, the generation of the trapped species (coumarin + ·OH → 7-hydroxycoumain) is improved by 2.2 and 2.6 times for def-WO_3_ and Cu_0.029_-def-WO_3_ over a 30 min reaction, confirming improved ·OH radicals due to the synergy between Cu single atoms and Ov. Such trend is consistent with the in situ EPR under light. As ·OH could activate CH_4_ to produce methyl radical (·CH_3_) (·OH + CH_4_ → ·CH_3_ + H_2_O), a higher amount of ·OH produced would be more beneficial to CH_4_ activation. Accordingly, both ·OOH and ·OH radicals are the reactive oxygen species during the photocatalytic CH_4_ conversion.

Initially, individual reactant adsorption was also simulated on the optimised photocatalysts. For oxygen adsorption on the Cu-def-WO_3_, simulation results reveal that the O_2_ bond length is largely stretched to 1.41 Å in the molecular form on the Cu (Fig. S25). Due to the low oxidation states of the Cu^δ+^, this stretched O_2_ is further readily protonated to ·OOH. Further, water adsorption on the W^δ+^ site near an oxygen vacancy was compared on the optimised Cu-def-WO_3_ and the pristine WO_3_ surface (Fig. S26). The calculated free reaction energy is −0.78 eV and −0.65 eV, respectively, which indicates that both Cu and W^δ+^ enhance the interaction with water. The W^δ+^ with lower oxidation states (*δ* < 6) near the O_v_ defect is ready to host the lone-pair electrons from the water. Thus, both water and W^δ+^ present near the O_v_ is the key to providing hole attraction and reaction sites for CH_4_ activation. Since CH_4_ could competitively react on the electron trapping site (Cu) or the hole trapping site (W^δ+^), the calculated free energy of the reaction is −0.04 and −0.43 eV on these two sites, respectively, as shown in the optimised geometries (Fig. S27), which indicates W^δ+^ site is the active site for methane activation. Nevertheless, it is still more difficult for CH_4_ to be activated on W^δ+^ compared with H_2_O due to the much smaller free reaction energy of water (−0.78 eV), which then indicates CH_4_ would be more likely to be directly activated by the ·OH radicals, rather than the photo-holes, through H_2_O oxidation with the photo-holes (Figs. S28 and S29).

Oxygen sources of HCHO were further investigated through the isotopic experiment with ^18^O_2_ and H_2_^18^O, separately. To confirm O_2_ as the oxygen source, 1 bar ^18^O_2_ and 19 bar CH_4_ over 20 mg Cu_0.029_-def-WO_3_ with 3 mL H_2_O were utilised for a 6-h reaction. GC-MS results (Fig. 4f) clearly show that the detected HCHO is composed of 81% HCH^16^O and 19% HCH^18^O, suggesting both O_2_ and H_2_O are the oxygen sources for HCHO production. Meanwhile, a much higher content of HCH^16^O suggests H_2_O serves as the major oxygen source. Photocatalytic CH_4_ conversion in the presence of isotopic labelled H_2_^18^O further supports the primary role of H_2_O on HCHO production due to the reversed composition of 20% HCH^16^O and 80% HCH^18^O. The participation of H_2_O in HCHO formation suggests that the ·OH radicals formed from the oxidation of H_2_O with photo-induced holes (H_2_O + h^+^ → ·OH + H^+^) could directly react with CH_4_ or ·CH_3_ to form _*_CH_3_OH (“_*_” indicates it’s still adsorbed), and then to HCHO. Isotopic experiments were also conducted with 5 bar ^13^CH_4_ and 1 bar O_2_ over 20 mg Cu_0.029_-def-WO_3_ in 3 mL H_2_O for a 6-h reaction. ^13^C nuclear magnetic resonance (NMR) spectroscopy in Fig. S30 shows only one signal at 82.2 ppm, assigned to HO^13^CH_2_OH, which was the diol structure of H^13^CHO in an aqueous solution (H^13^CHO + H_2_O → HO^13^CH_2_OH)^18,20^. This result confirms that the carbon source of the produced HCHO comes from CH_4_. Moreover, it also confirms the high selectivity with no CH_3_OH and HCOOH production, consistent with the ^1^H NMR results (Fig. S31).

**Fig. 5:**
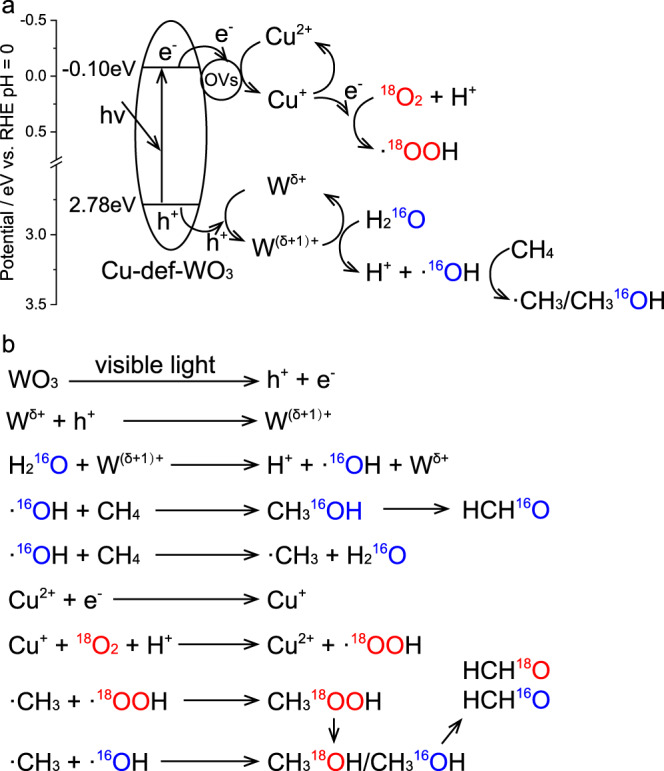
Proposed mechanism. **a**, **b** Schematic of charge transfer steps during selective CH_4_ oxidation over Cu_0.029_-def-WO_3_.

Based on the above results, particularly based on the strong evidence of the characteristics of the ·OH and ·OOH radicals by in situ EPR, a radical mechanism (Fig. 5a, b) of aerobic photocatalytic CH_4_ conversion to HCHO is proposed here over the Cu_0.029_-def-WO_3_ catalyst, which consists of nanocrystalline WO_3_ substrate with single atoms Cu^δ+^ and O_v_ /(W^δ+^). In general, under visible-light irradiation, electrons are excited from the valence band (VB) to the conduction band (CB) of WO_3_. The active electrons then migrate to Cu^2+^ to form Cu^+^ (Cu^2+^-e^-^). The adsorbed O_2_ on the Cu site next reacts with the electrons trapped by Cu^+^ to form the ·OOH radical, which requires a potential of −0.046 eV [63]. In parallel, photo-induced holes are trapped by the W^δ+^ with low oxidation (W^δ+^ + h^+^ → W^(δ+1)+^), and then react with the chemical adsorbed H_2_O at the oxygen vacancy induced W^δ+^ site to form ·OH radicals. Meanwhile, the ·OH radical could directly activate CH_4_ and then react with ·CH_3_ to form _*_CH_3_OH intermediate, then being transformed to the desired HCHO. The absence of CH_3_OOH and CH_3_OH in ^1^H NMR spectra (Fig. S32) may be attributed to the immediate conversion of such intermediates to HCHO. On the basis of efficient activation of the different reactants (H_2_O, O_2_ and CH_4_) with the assistance of the specific synergy of O_v_ induced W^δ+^ site and highly dispersed Cu atomic co-catalyst, the selectivity of HCHO over Cu_0.029_-def-WO_3_ was successfully maintained at nearly 100% over a long period of time to 10 h.

In summary, an effective strategy to overcome the dilemma of enhancing the efficient activation while suppressing the over-oxidation of CH_4_ has been developed for the selective production of value-added HCHO over a noble-metal-free Cu_0.029_-def-WO_3_ photocatalyst. The WO_3_ substrate provides the visible-light responsive activity for CH_4_ conversion, while the atomically dispersed Cu acts as an effective electron acceptor, as indicated from the analysis of the in situ XPS and EPR spectra under light irradiation. O_v_ induce the formation of reactive W^δ+^ species, which further enhances the selective chemical adsorption and activation of H_2_O. The coordinated ensemble of the dual active sites synergistically leads to efficient charge separation and transfer. As a result, under visible-light irradiation at room temperature, a superior photocatalytic CH_4_ conversion efficiency has been achieved with a high TOF of 8.5 × 10^6^ μmol_(HCHO)_·g^−1^_(cocatalyst)_·h^−1^, out-performing previously reported photocatalysts, even much better than the noble-metal photocatalytic processes^19^. Nearly 100% selectivity and a high HCHO evolution rate of 4979.0 μmol·g^−1^ have been achieved under 420 nm light irradiation at room temperature. Isotopic experiments provide strong evidence that both O_2_ and H_2_O are the oxygen sources for HCHO production, with H_2_O being the major one. This work provides a new avenue for simultaneous regulation of CH_4_ activation under visible-light irradiation and suppression of over-oxidation by incorporation of adjacent dual active sites, which is of great interest in net-zero green conversion and upgrading of natural resources to high-value chemicals.

## Methods

### Preparation of Cu_*x*_-def-WO_3_

Cu_*x*_-def-WO_3_ photocatalysts were prepared by a highly reproducible incipient impregnation method and subsequent hydrogen reduction. In a typical experiment, 5.0 g WO_3_ was first mixed with 2.0 mL CuCl_2_ aqueous solution containing a certain amount of CuCl_2_. After uniformly stirring and ageing at ambient temperature overnight, the obtained solid was dried at 60 ^o^C for another 12 h. The dried samples were then grounded and calcined at 250 ^o^C for 2 h at a ramping rate of 5 ^o^C/min in 5 vol.% H_2_/Ar flow (80 mL/min). The as-prepared samples were denoted Cu_*x*_-def-WO_3_, where *x*% represented the mass percentage of the copper atom. For comparison, the defective WO_3–*x*_ (denoted def-WO_3_) was prepared under identical conditions but without CuCl_2_ addition.

### Characterisations

XRD patterns were acquired on the *D8 ADVANCE* diffractometer (*Bruker Co., Ltd*). Raman spectra were acquired on the *DXR 2DXR2* instrument (*Thermo Fisher Scientific, Co., Ltd*). XPS spectra were measured with the *PHI 5000 Versa Probe III* instrument (*ULVAC-PHI Co., Ltd*). In situ XPS spectra were collected in dark or under visible-light irradiation for 20 min on the *Thermo ESCALAB 250Xi* instrument with an Al Kα radiation source. In situ solid-state EPR spectra were measured on the *ELEXSYS II* EPR instrument with 20 mg photocatalyst in dark or under 420 nm light irradiation. HRTEM images were captured on the *Talos F200X* instrument (*FEI Co., Ltd*). Where photocatalyst was pre-dispersed under sonication and dipped on the molybdenum mesh as the copper-free supporting substrate. UV–vis DRS spectra were recorded on the *UV-3600 Plus* spectrometer (*Shimadzu Co., Ltd*) with spectroscopic pure BaSO_4_ as the references. Steady-state and time-decay PL spectra were recorded at room temperature on the QM400 and FLSP920 spectrofluorometers, respectively.

### Photocatalytic methane conversion

Photocatalytic CH_4_ conversion reaction was conducted in the 200 mL stainless-steel high-pressure reactor equipped with Teflon lining. Typically, 5 mg photocatalyst was first dispersed in 120 mL distilled H_2_O under stirring. The reactor was then sealed and purged with ultrapure oxygen (99.99 vol.%) under moderate stirring for about 20 min to remove the air completely. After acquiring atmospheric oxygen or desired partial pressure, ultrapure CH_4_ (99.99 vol.%) was injected into the reactor to achieve total pressure of 20 bar. The reactor with top-irradiation was then irradiated with the LED lamp source (420 nm, *PLS-LED100C*, *Beijing Perfectlight Technology Co., Ltd*). Photocatalytic reaction was conducted for 2 h at 25 ^o^C. The gaseous products were measured by gas chromatograph (*GC2014*, *Shimadzu Co., Ltd*) equipped with a thermal conductivity detector and flame ionisation detector. The dissolved CO_2_ was also analysed through the precipitation method with excessive Ba(OH)_2_, where excess Ba(OH)_2_ was added into 10 mL supernate after reaction^67^. No discernible precipitation was observed, indicating the dissolved CO_2_ was too low to be measured. The possible existence of other products, including CH_3_OOH, CH_3_OH and HCOOH, was analysed by ^1^H nuclear magnetic resonance spectroscopy (*AVANCE III, JEOL Ltd*). The results (Fig. S31) confirmed that no CH_3_OOH, CH_3_OH and HCOOH were produced during the CH_4_ conversion reaction by the photocatalyst. HCHO was measured by the colorimetric method based on the reaction between acetylacetone and HCHO in the presence of acetic acid and ammonium acetate^68^. A trace amount of CO_2_ was detected during all photocatalytic reactions, demonstrating the ultrahigh HCHO selectivity.

Reusability of Cu_0.029_-def-WO_3_ was tested by photocatalytic CH_4_ conversion. Parallel experiments was conducted under identical conditions to replenish the losses of photocatalyst. After each experiment, the reactant was centrifuged and dried at 60 ^o^C in vacuum. Then 5 mg collected photocatalyst was re-used for the next cycling experiment.

### Isotopic experiments

For carbon source investigation with isotopic labelled ^13^CH_4_: 20 mg Cu_0.029_-def-WO_3_ photocatalyst was dispersed in 3 mL H_2_O, then the reactor was vacuumed for 30 min and refilled with 1 bar O_2_ and 5 bar ^13^CH_4_. The reaction was conducted for 6 h to gain more concentrated products for detection facilitation. As H^13^CHO tended to exist as HO^13^CH_2_OH in aqueous solution, the products were identified by ^13^C NMR (*AVANCE III*, *JEOL Ltd*).

For oxygen source investigation with isotopically labelled ^18^O_2_: 20 mg Cu_0.029_-def-WO_3_ photocatalyst was dispersed in 3 mL H_2_O, then the reactor was vacuumed for 30 min and refilled with 1 bar ^18^O_2_ and 19 bar CH_4_. The reaction was conducted for 6 h to gain more concentrated products for detection facilitation. The as-produced HCH^18^O was measured with GC-MS (*QP2020, Shimadzu Co., Ltd*) equipped with the Cap WAX column to identify the existence of H_2_^18^O.

For oxygen source investigation with isotopic labelled H_2_^18^O: 20 mg Cu_0.029_-def-WO_3_ photocatalyst was dispersed in 3 mL H_2_O, then the reactor was vacuumed for 30 min and refilled with 1 bar ^18^O_2_ and 19 bar CH_4_. The reaction was conducted for 6 h to gain more concentrated products for detection facilitation. The as-produced HCH^18^O was measured with GC-MS (*QP2020, Shimadzu Co., Ltd*) equipped with the Cap WAX column to identify the existence of H_2_^18^O.

### Photoelectrochemical measurements

Mott-Schottky plots, electrochemical impedance spectroscopy (EIS) and photocurrent density were measured on the three-electrode system with an electronic workstation (*CHI660E*). Photocatalyst coated by indium tin oxide (ITO) glass (10 × 10 mm), Ag/AgCl electrode and platinum sheet electrode were respectively employed for working, reference and counter electrodes, with 0.1 mol·L^−1^ Na_2_SO_4_ solution as the electrolyte. The working electrodes were prepared by scraping the paste-like mixture containing 100 mg of different photocatalysts, 450 μL ethanol and 50 μL Nafion solution (*Shanghai Adamas Reagent Co., Ltd*). Before measurement, the working electrodes were dried at 60 °C in vacuum.

### Monitor of the reactive oxygen species

5, 5-dimethyl-1-pyrroline N-oxide (DMPO) was used as the trapping agent for the monitoring of the reactive oxygen species, including hydroperoxyl (·OOH) and hydroxyl (·OH) radicals. For monitoring the generation of ·OOH radical, 10 mg Cu_0.029_-def-WO_3_ photocatalyst was dispersed in 5 mL methanol in the dark and purged with ultrapure O_2_ (99.99 vol.%) for 20 min. For monitoring the generation of ·OH radical, 5 mg Cu_0.029_-def-WO_3_ photocatalyst was dispersed in 5 mL distilled H_2_O in the dark and purged with ultrapure argon (99.99 vol.%) for 20 min. In situ EPR spectra of the above suspension in dark and under light were then obtained on the *ELEXSYS II* EPR instrument.

### Analysis the productivity of ·OH radical

Coumarin was used as the probe to evaluate the productivity of ·OH radical due to the reaction between coumarin and ·OH to produce 7-hydroxycoumain (7HC). Typically, 20 mg Cu_0.029_-def-WO_3_ photocatalyst was dispersed in 100 mL aqueous coumarin solution (0.5 mM). After stirring in dark for 30 min, 5 mL reactant was sampled every 5 min under 420 nm light irradiation. After filtration, the PL spectra of the formed 7HC were measured by *F4500* spectrofluorometer.

### Theoretical calculations

All the calculations were performed based on density functional theory (DFT), implemented in the Vienna ab initio Package (VASP)^69^. Electron-ion interactions were described with the projector-augmented-wave (PAW) method with an energy cut-off set to 500 eV^70^. The PBE functional was used to optimise the structural models and to analyse the electronic structures^71^. The structures were fully relaxed until the changes in energy and in the force upon ionic displacement were no greater than 10^−5 ^eV and 0.02 eV/Å, respectively. The delocalised electron was treated by the (DFT + U) Dudarev approach with the effective *U* value set to 5 and 8 for tungsten and copper, respectively^72^. The *U* values are in line with previous calculations on WO_3_^73^. Due to the complex oxidation states of Cu in Cu-def-WO_3_, we have adopted the *U* value from one report^74^, as this *U* value has been well assessed for Cu_2_O, CuO and Cu_3_O_4_. The partial charges were determined using the Bader charge analysis^75^. The chemical potential of Cu single atom is derived from the bulk copper. The entropy of gas phase H_2_O, O_2_, HCHO are obtained from the NIST database under standard conditions^76^.

The adsorption energy (*E*_ad_) is calculated by

2 $${E}_{{{{{{{\mathrm{ad}}}}}}}}={E}_{{{{{{{\mathrm{total}}}}}}}}-{E}_{{{{{{{\mathrm{adsorbent}}}}}}}}-{E}_{{{{{{{\mathrm{sub}}}}}}}}$$

where the *E*_total_, *E*_adsorbent_ and *E*_sub_ is the total energy of the system, the energy of the adsorbent and the substrate, respectively.

The reaction free energy is calculated according the following equation:

3 $$\triangle G={E}_{{{{{{{\mathrm{ad}}}}}}}}+\triangle {{{{{{\mathrm{ZPE}}}}}}}-T\triangle S$$

where the *E*_ad_ is the adsorption energy, ΔZPE is the changes of the zero point energy, Δ*S* is the changes of the entropy of the reaction. The internal thermal energy *U*_0→*T*_ is calculated based on

4 $${U}_{0\to T}=R{T}^{2}{\left(\frac{\delta {{{{{\rm{ln}}}}}}q}{\delta T}\right)}_{V}$$

The reaction temperature is set to 300 K.

## Supplementary information

Supplementary Information

## Data Availability

The data that support the findings of this study are available from the corresponding author upon reasonable request. Source data are provided with this paper.

## Source data

Source Data
