# Supplementary material for: Nearly 100% selective and visible-light-driven methane conversion to formaldehyde via. single-atom Cu and Wδ+
Source: Nat Commun. 2023 May 10;14:2690. doi: 10.1038/s41467-023-38334-7 (PMC10172301; doi:10.1038/s41467-023-38334-7)
Supplement: Supplementary file 1 — Supplementary Information [file 41467_2023_38334_MOESM1_ESM.pdf]

Supporting Information for

Nearly 100% Selective and Visible-Light-Driven Methane Conversion to Formaldehyde via. Single-Atom Cu and $W^{\delta+}$

Lei Luo[1,†], Xiaoyu Han[2], Keran Wang[1], Youxun Xu[3], Lunqiao Xiong[3], Jiani Ma[1], Zhengxiao Guo[4*], Junwang Tang[3*]

1 Key Lab of Synthetic and Natural Functional Molecule Chemistry of Ministry of Education, the Energy and Catalysis Hub, College of Chemistry and Materials Science, Northwest University, Xi'an 710127, P. R. China.

2 Department of Chemistry, The University of Manchester, Manchester M13 9PL, U.K

3 Department of Chemical Engineering, University College London, Torrington Place, London WC1E 7JE, UK.

4 Department of Chemistry, The University of Hong Kong, Pokfulam Road, Hong Kong 999077, P. R. China.

† Present Address: State Key Laboratory of Catalysis, Dalian Institute of Chemical Physics, The Collaborative Innovation Centre of Chemistry for Energy Materials (iChEM), Dalian National Laboratory for Clean Energy, Chinese Academy of Sciences, Zhongshan Road 457, Dalian 116023, P. R. China.

* Corresponding author.

E-mail: zxguo@hku.hk

E-mail: junwang.tang@ucl.ac.uk

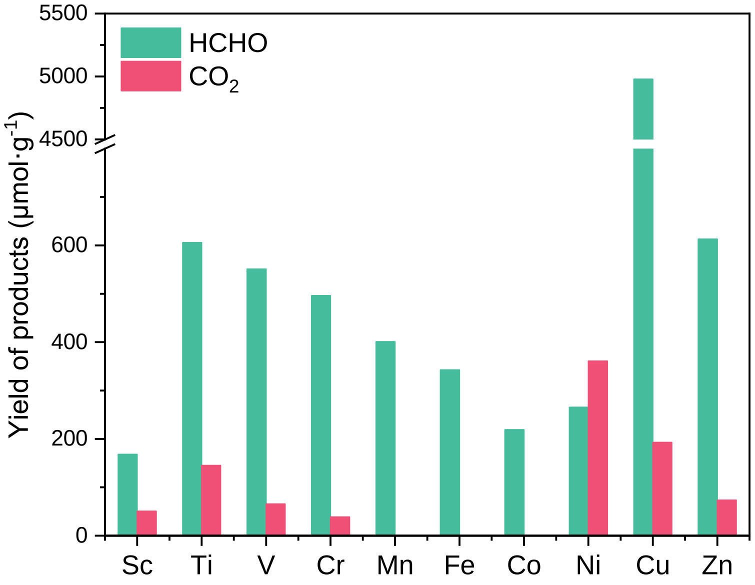

Figure S1. Photocatalytic methane conversion performances of M-def-WO_3. M represent all 3d transition metals including Sc, Ti, V, Cr, Mn, Fe, Co, Ni, Cu and Zn.

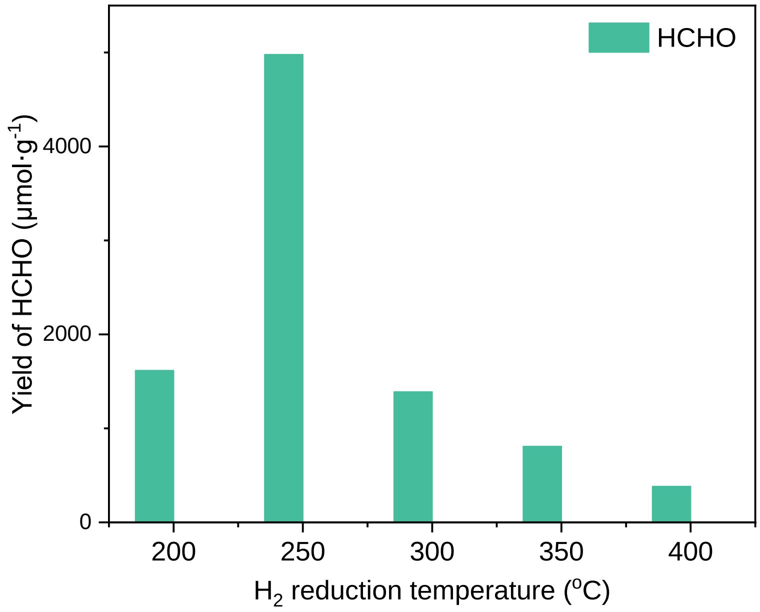

Figure S2. Photocatalytic methane conversion performances of $Cu_{0.029}$-def-WO_3 prepared by thermal hydrogen reduction at different temperature.

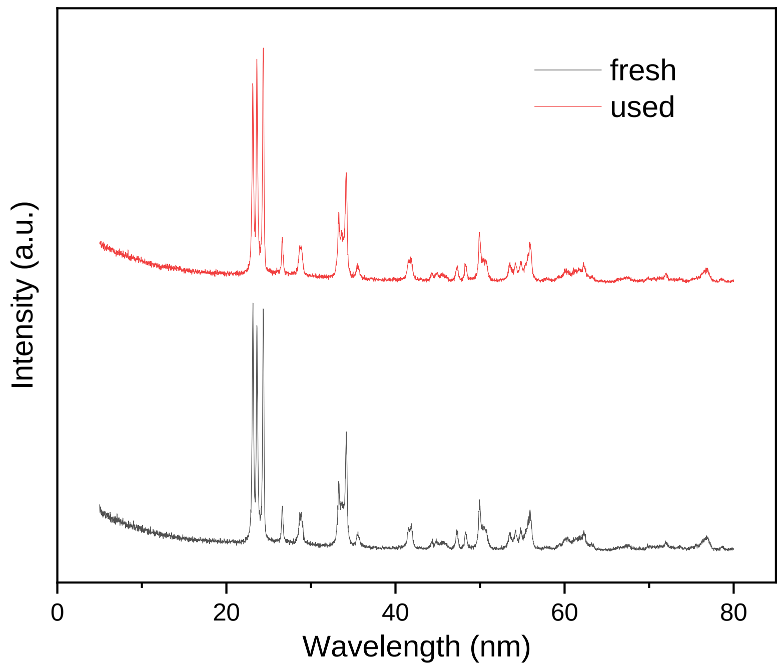

Figure S3. XRD patterns of the fresh prepared and used $Cu_{0.029}$-def-WO_3 photocatalyst.

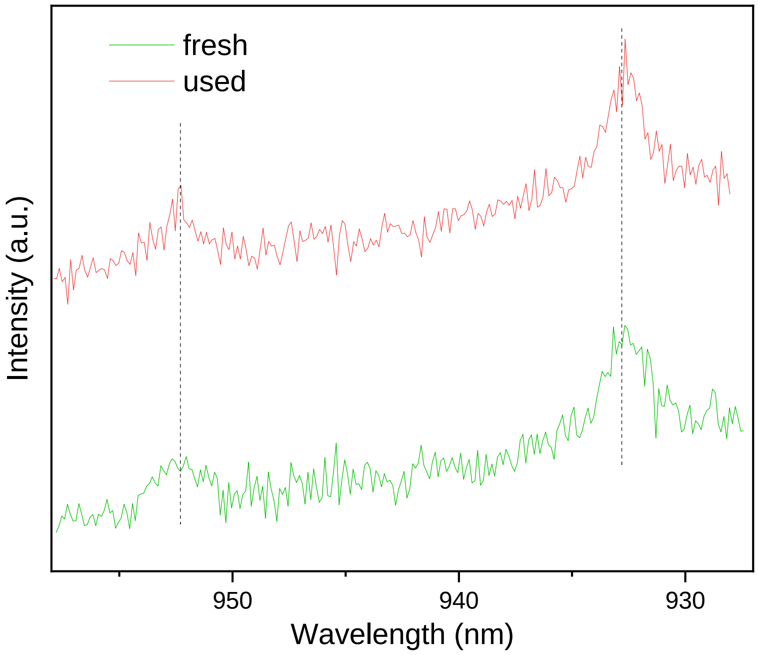

Figure S4. High-resolution XPS spectra of the fresh and used $Cu_{0.029}$-def-WO_3 photocatalyst.

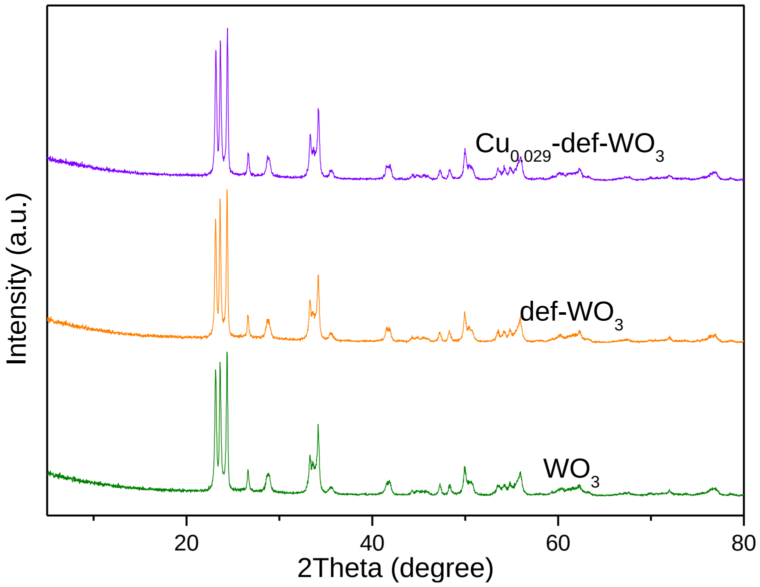

Figure S5. XRD patterns of WO_3, def-WO_3 and $Cu_{0.029}$-def-WO_3.

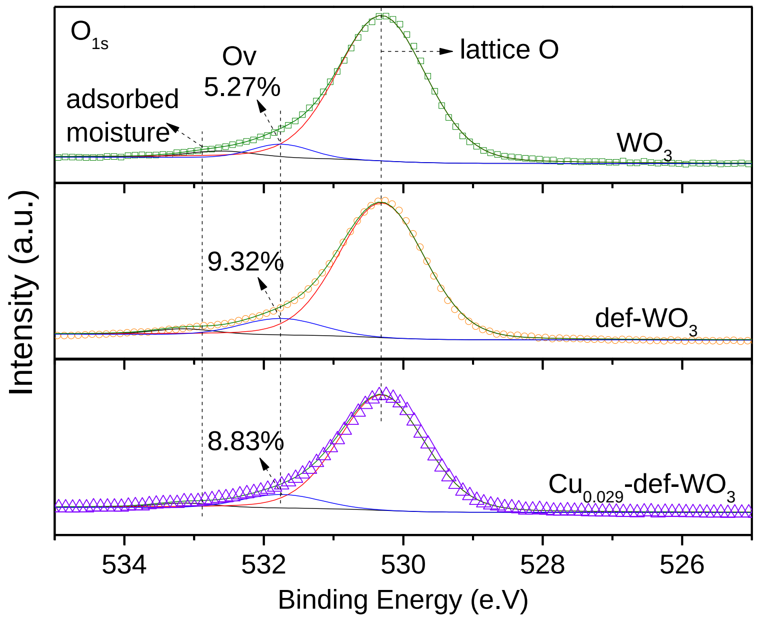

Figure S6. High-resolution O_{1s} XPS spectra of WO_3, def-WO_3 and $Cu_{0.029}$-def-WO_3.

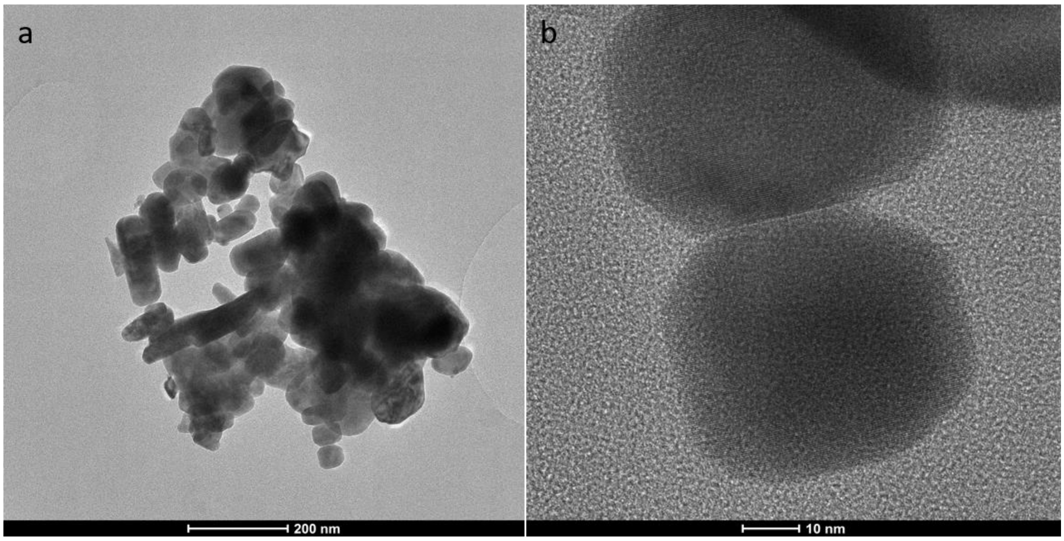

Figure S7. (a, b) TEM images of pristine WO_3.

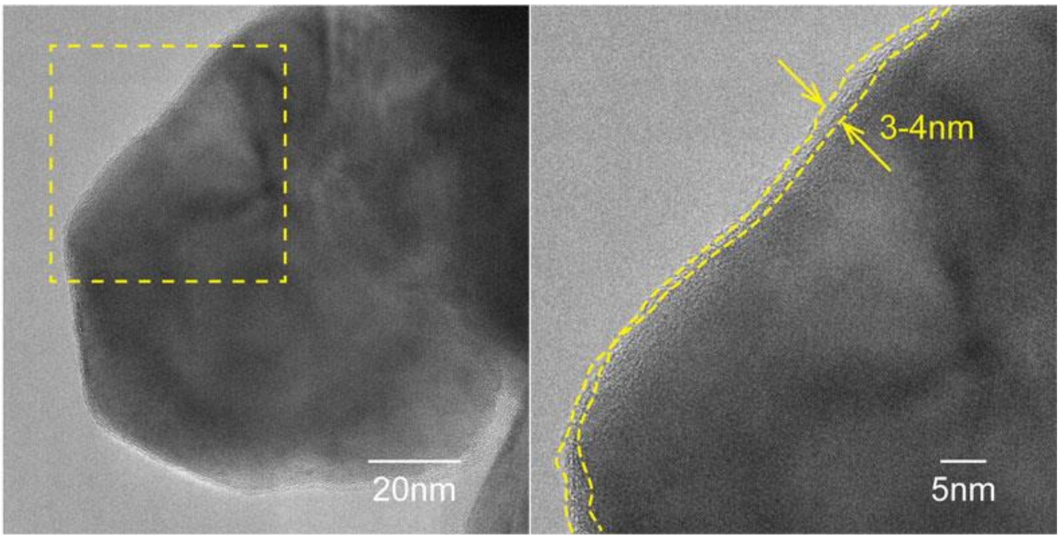

Figure S8. TEM image of (a, b) def-WO_3.

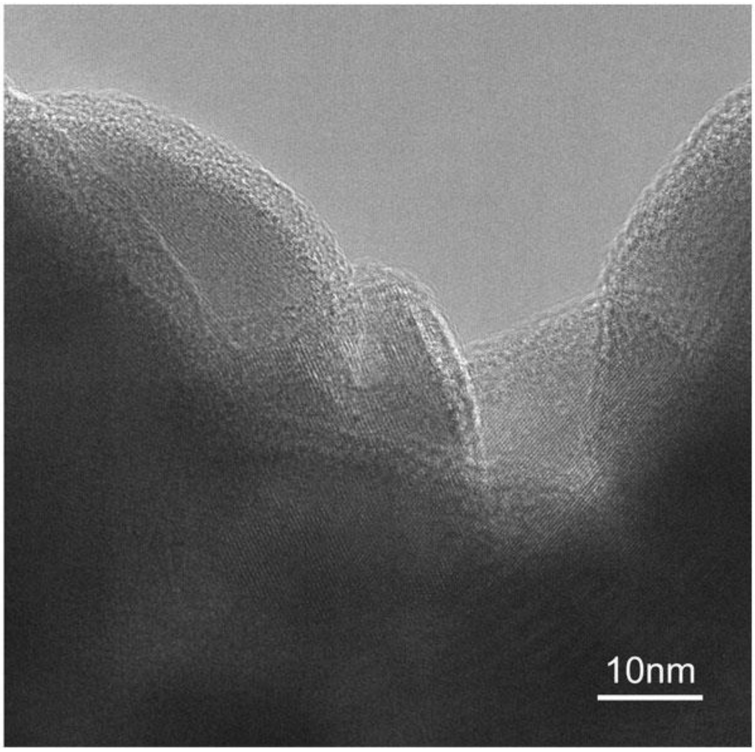

Figure S9. TEM image of Cu-def-WO_3.

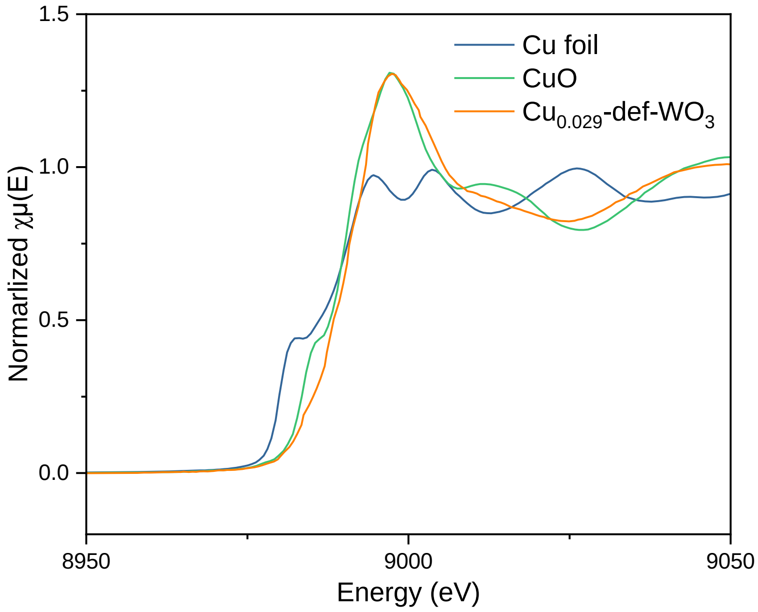

Figure S10. Normalized XANES spectra of the Cu K-edge of $Cu_{0.029}$-def-WO_3, CuO and Cu-foil.

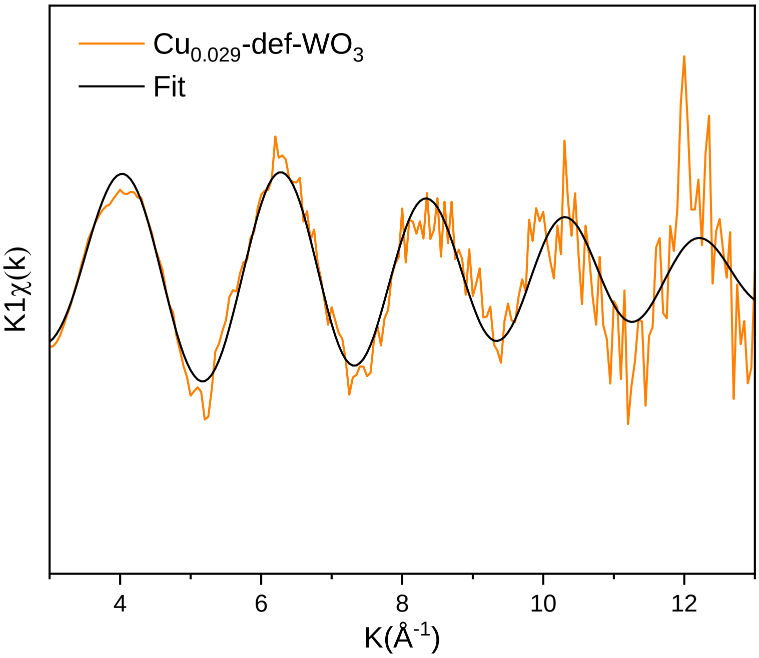

Figure S11. K^1-weighted raw EXAFS spectra at the Cu K-edge of sample $Cu_{0.029}$-def-WO_3.

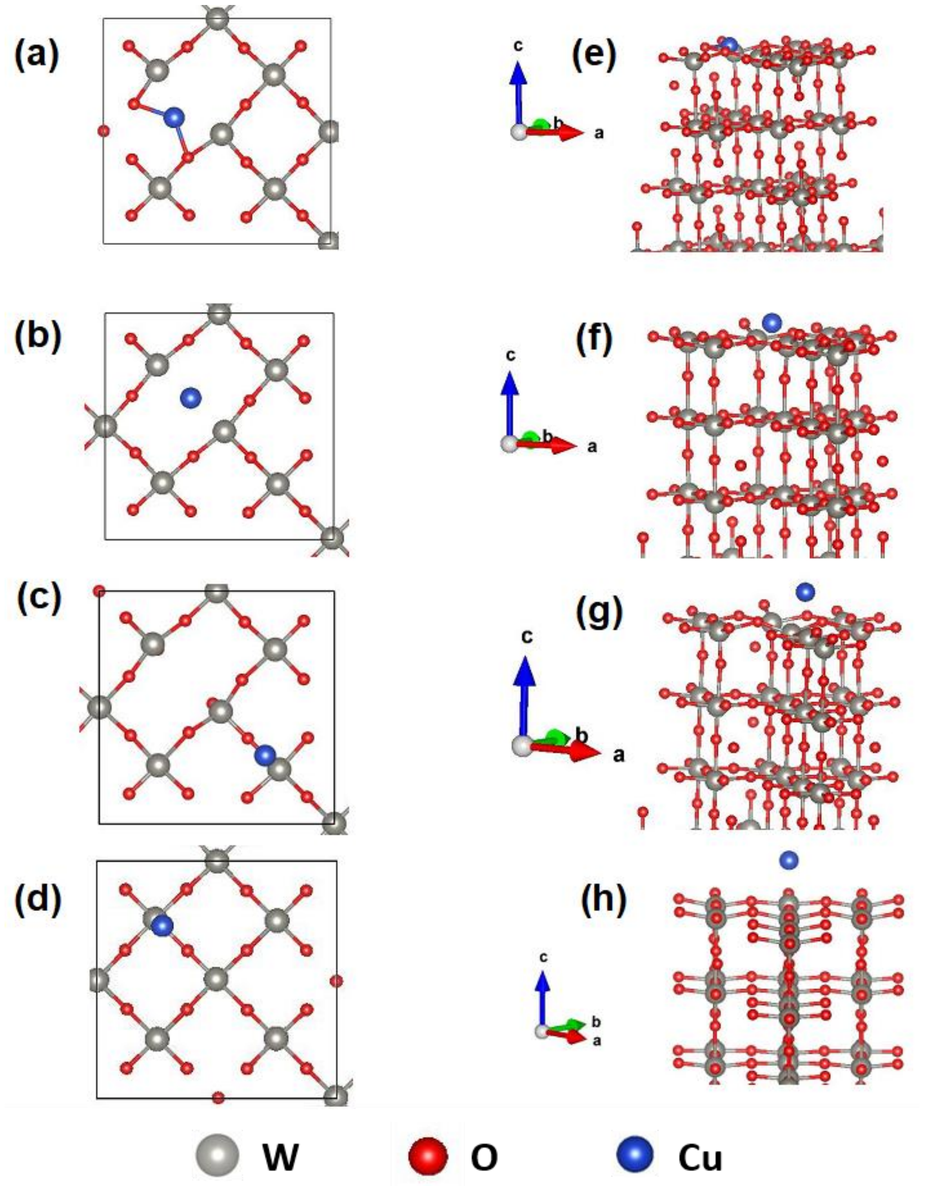

Figure S12. The optimised configurations of the atomic Cu at (a, e) A, (b, f) B, (c, g) C sites of def-WO_3 and (d, h) pristine WO_3 over the (002) surface, respectively with (a-d) the top views and (e-h) the corresponding side views.

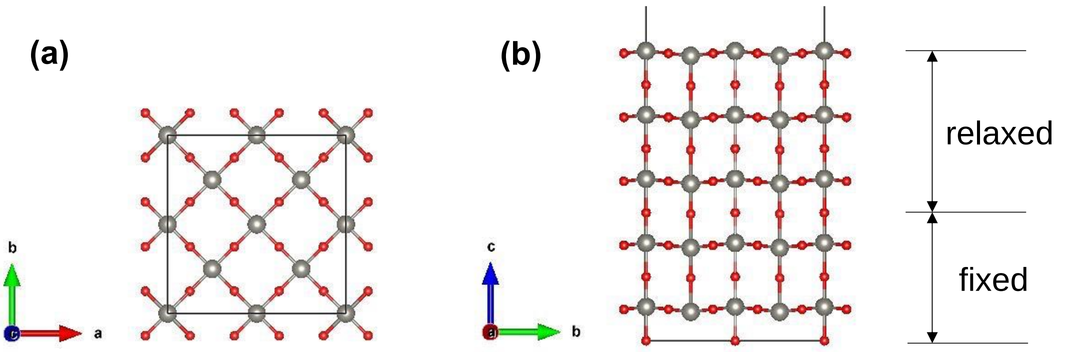

Figure S13. (a) The top view and (b) side view of the pristine WO_3 (002) surface model. The grey and red balls represent W atoms and O atoms, respectively.

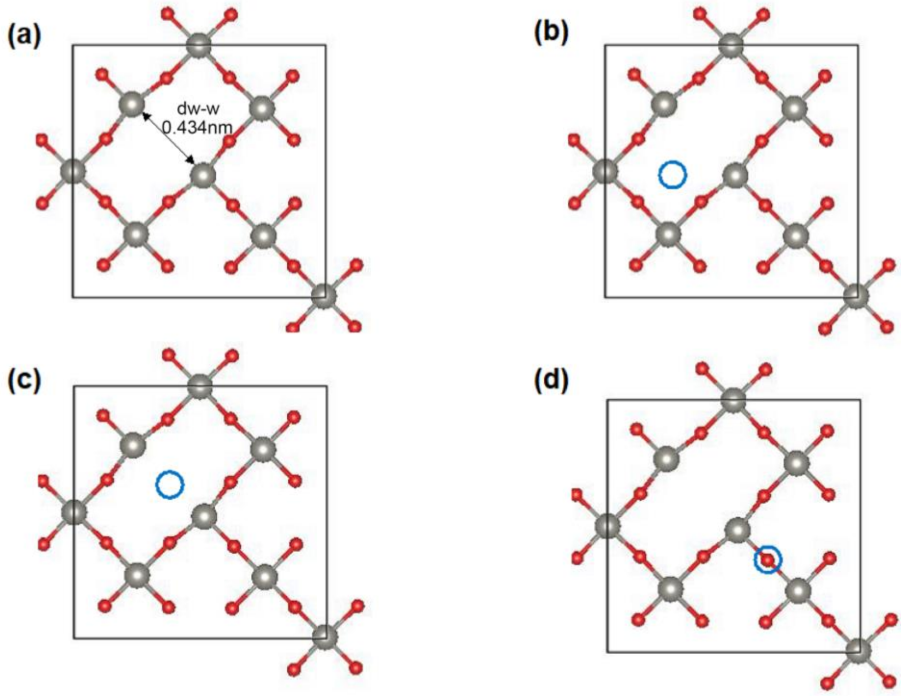

Figure S14. (a) The top view of the first layer of WO_3(002) surface with single O vacancy. Three possible adsorption sites of atomic Cu atom on (b) A site, (c) B site and (d) C site.

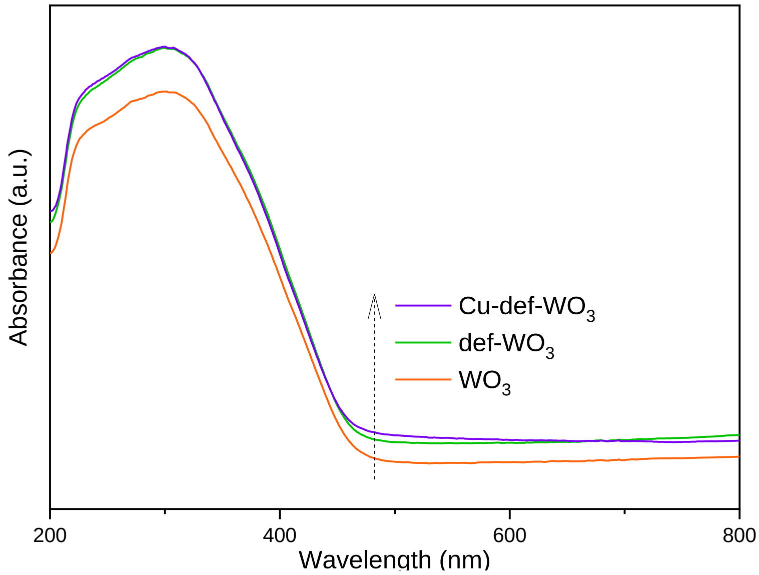

Figure S15. UV-vis diffraction spectra of WO_3, def-WO_3 and $Cu_{0.029}$-def-WO_3.

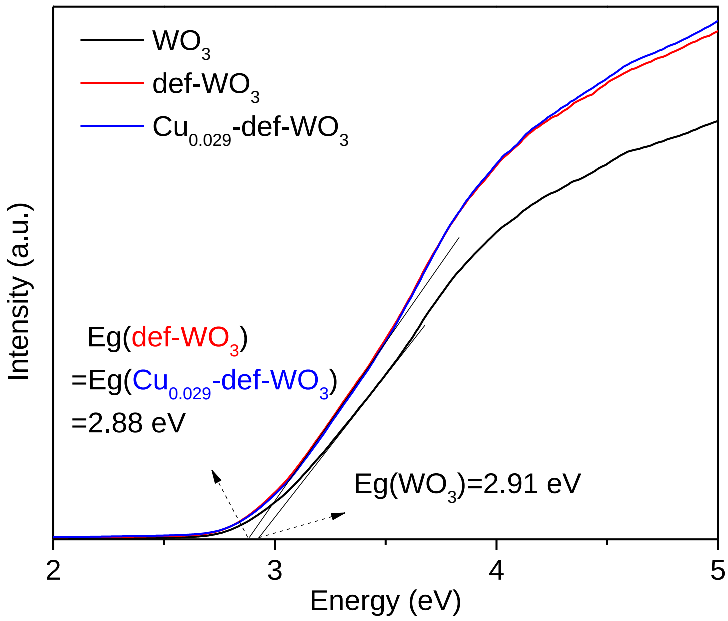

Figure S16. Tauc plots of WO_3, def-WO_3 and $Cu_{0.029}$-def-WO_3.

Bandgap energy was calculated using the Tauc plots (**Figure S15**) and by the following equation: $\alpha h\upsilon = A(h\upsilon\text{-}Eg)^n$, where A is a constant, hυ is the corresponding photon energy, α is the absorption coefficient, and n depends on the type of the optical transition. The plot with linear extrapolation to the photon energy gives the band gap energy.

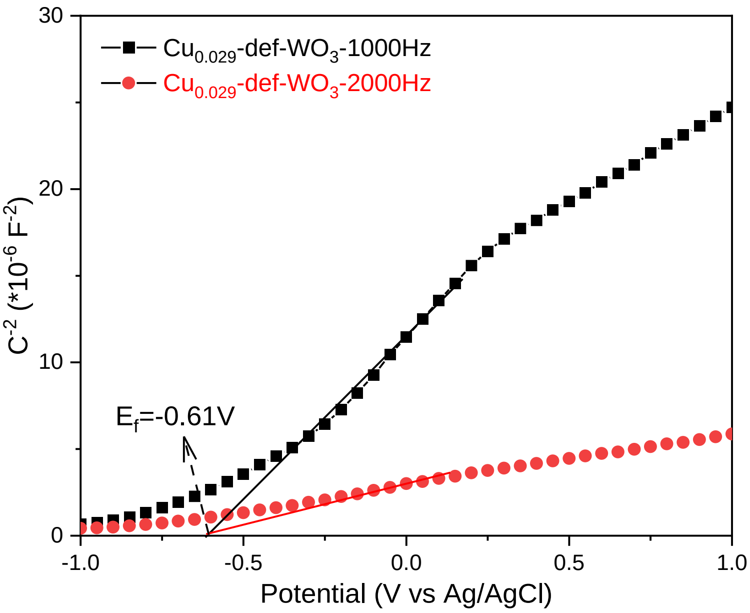

Figure S17. Mott-Schottky plots of $Cu_{0.029}$-def-WO_3 measured at pH=7.

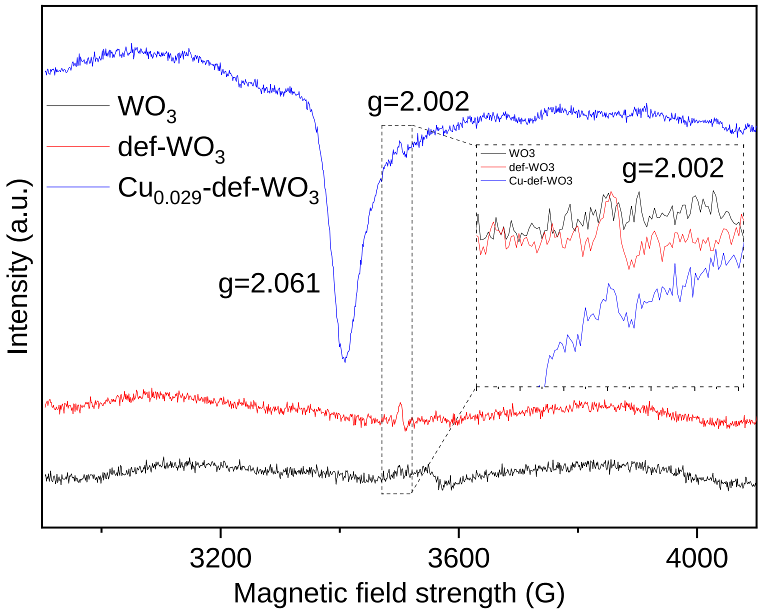

Figure S18. EPR spectra of WO_3, def-WO_3 and $Cu_{0.029}$-def-WO_3 photocatalysts in dark. The insert is the normalized and enlarged region between 3440 G and 3560 G (the dotted line rectangle) using the intensity at 4100 G as the reference, where the peak at g = 2.002 is attributed to the spin-electrons trapped by the oxygen vacancies.

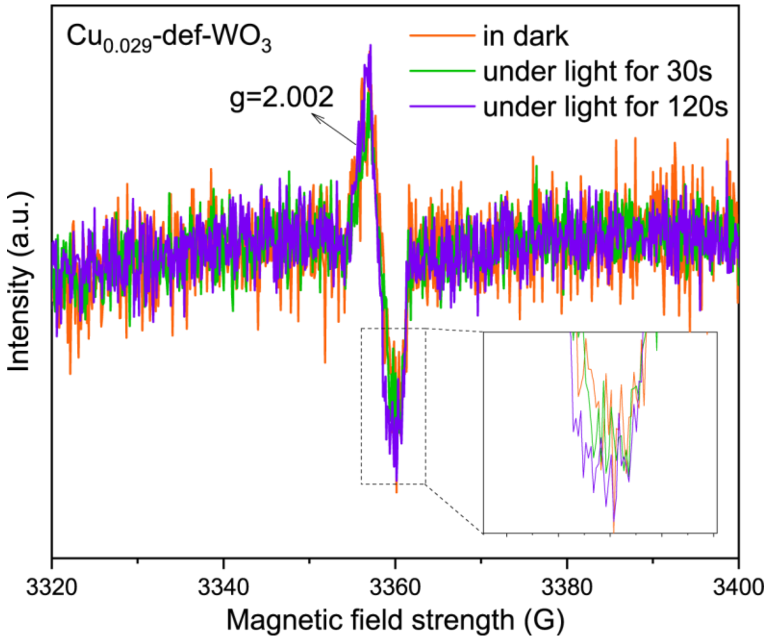

Figure S19. Low-temperature *in-situ* solid-state EPR spectra of $Cu_{0.029}$-def-WO_3 under 420 nm irradiation for different time.

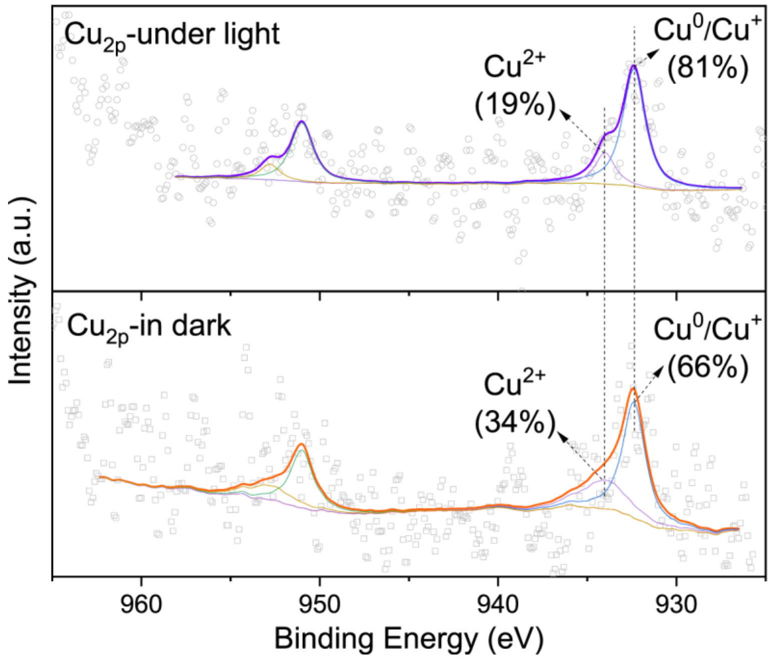

Figure S20. *In-situ* high-resolution Cu_{2p} XPS spectra of $Cu_{0.029}$-def-WO_3 under 420 nm light irradiation.

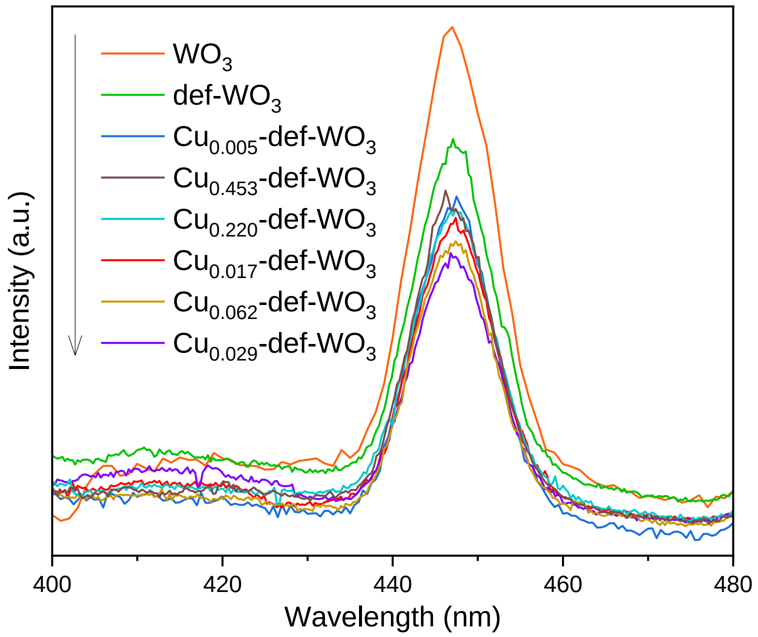

Figure S21. Steady-state PL spectra of different photocatalysts.

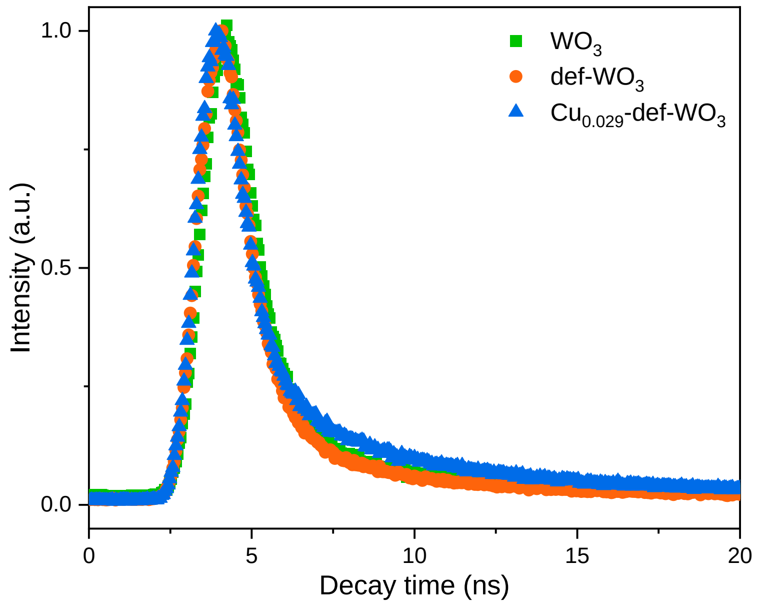

Figure S22. Time-decay PL spectra of WO_3, def-WO_3 and $Cu_{0.029}$-def-WO_3.

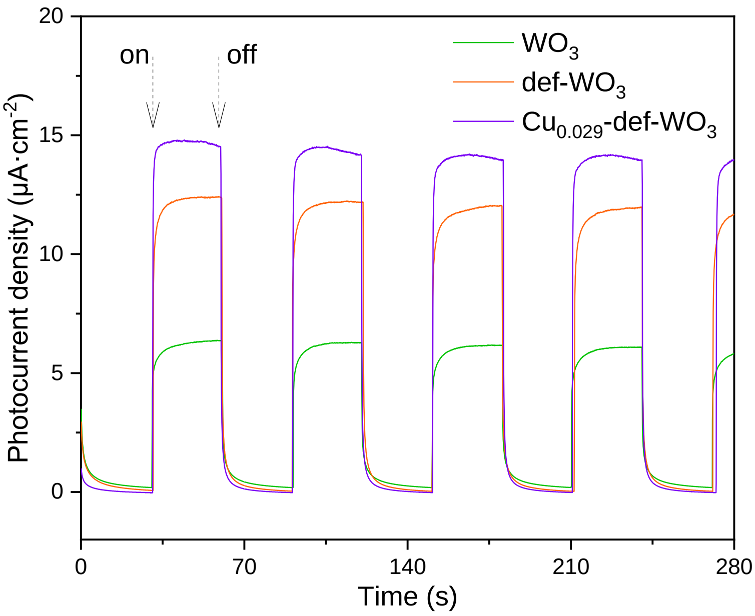

Figure S23. Photocurrent density of WO_3, def-WO_3 and $Cu_{0.029}$-def-WO_3.

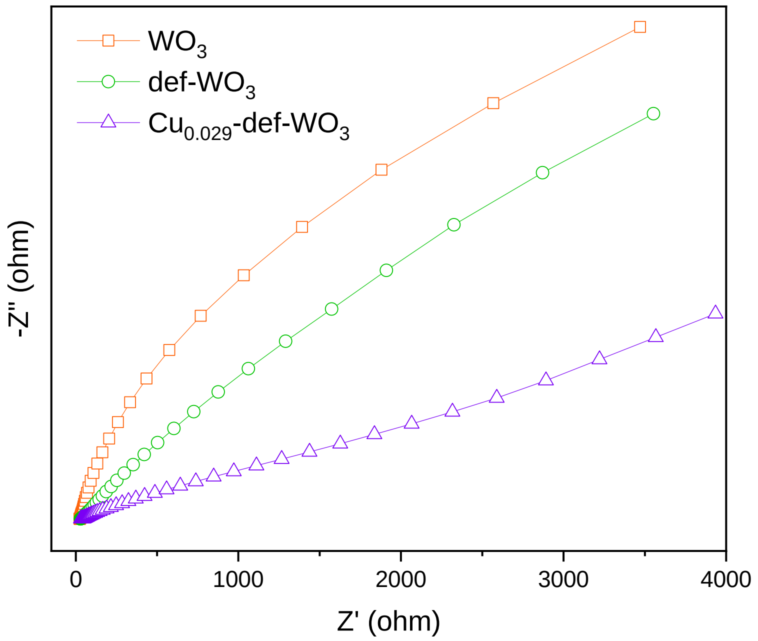

Figure S24. EIS plots of WO_3, def-WO_3 and $Cu_{0.029}$-def-WO_3.

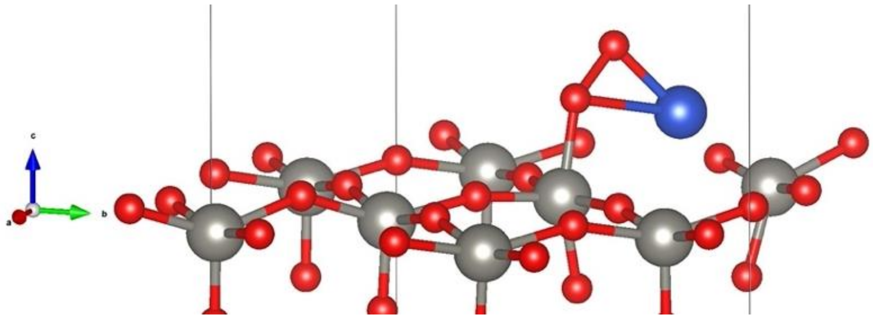

Figure S25. The optimised configuration of O_2 adsorption on Cu-def-WO_3.

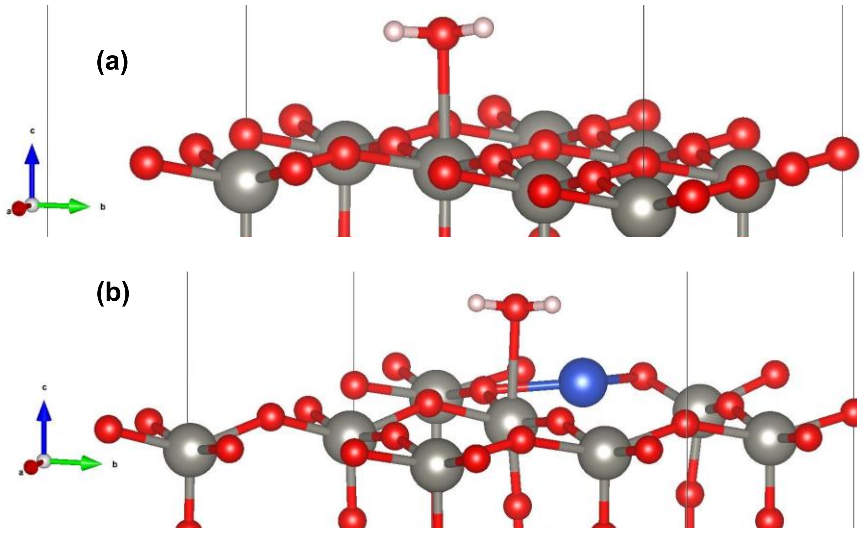

Figure S26. The optimised configuration of water adsorption on (a) pristine WO_3 surface and (b) the optimised Cu-def-WO_3 surface.

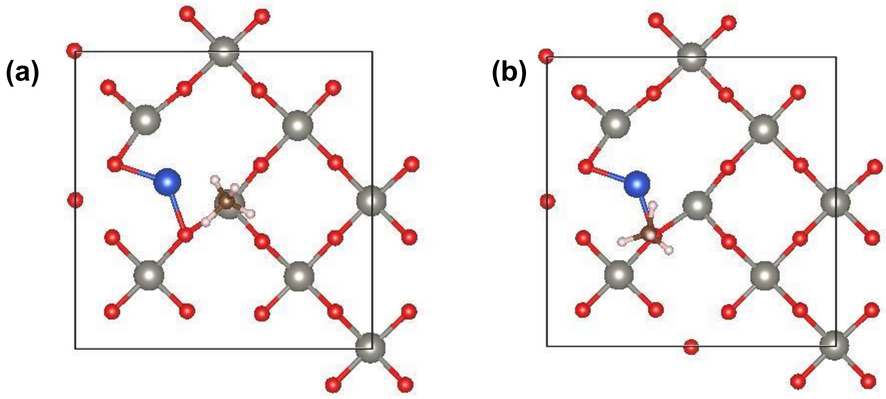

Figure S27. The optimised configuration of CH_4 adsorption on (a) hole trapping site ($W^{\delta+}$) and (b) electron trapping site ($Cu^{\delta+}$). The calculated free reaction energy is -0.04 and -0.43 eV on $W^{\delta+}$ and $Cu^{\delta+}$ sites, respectively.

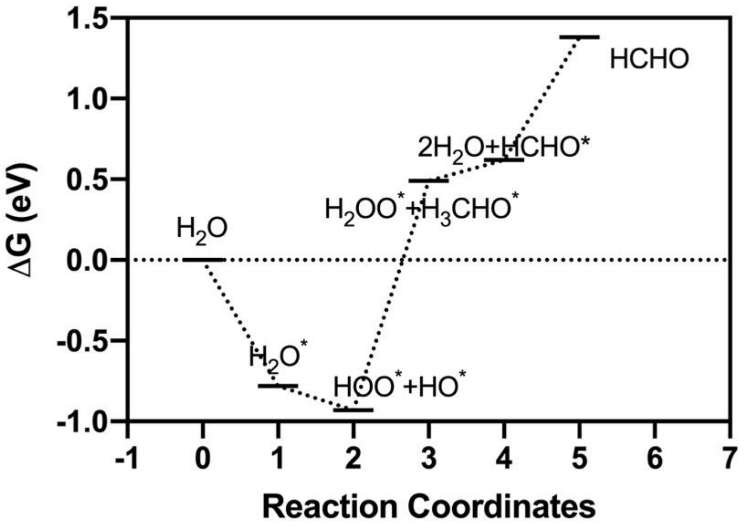

Figure S28. The calculated free energies of the proposed reaction pathway.

According to our experiment results, especially the GC-MS with isotopic labelled data illustrated, we proposed the possible reactions steps in the following and calculated the reaction free energy (**Figure S27**), where the * represents the adsorbed state at the reaction site. For the proposed reaction pathway, it is noted that the O_2 adsorption spontaneously split the water into ·H and·OH radicals on the surface to form ·OOH and ·OH, and the calculated ΔG_2 is -0.15 eV, which further confirms our findings in our monitored trapping agents. The optimised configuration is shown in **Figure S28** with labelled Bader effective charges. The Bader analysis shows that the O in the hydroperoxide is significant reduced, which will drive the breakage of the CH bond in CH_4 in Step 3.

Step 1: $H_2O + * \rightarrow H_2O^*$

Step 2: $O_2 + H_2O^* \rightarrow HOO^* + HO^*$

Step 3: $HOO^* + HO^* + CH_4 \rightarrow H_2OO^* + H_3CHO^*$

Step 4: $H_2OO^* + H_3CHO^* \rightarrow 2H_2O + HCHO^*$

Step 5: $HCHO^* \rightarrow HCHO + *$

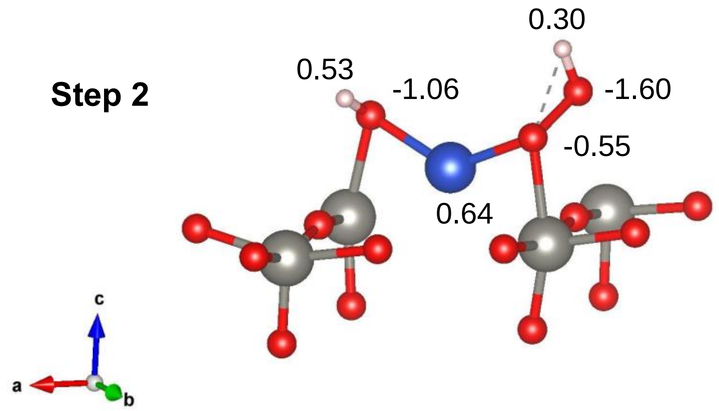

Figure S29. The optimised reaction Step 2 with O_2 and H_2O adsorption on Cu-def-WO_3 surface. The Bader effective charges of key elements are labelled on the atoms nearby.

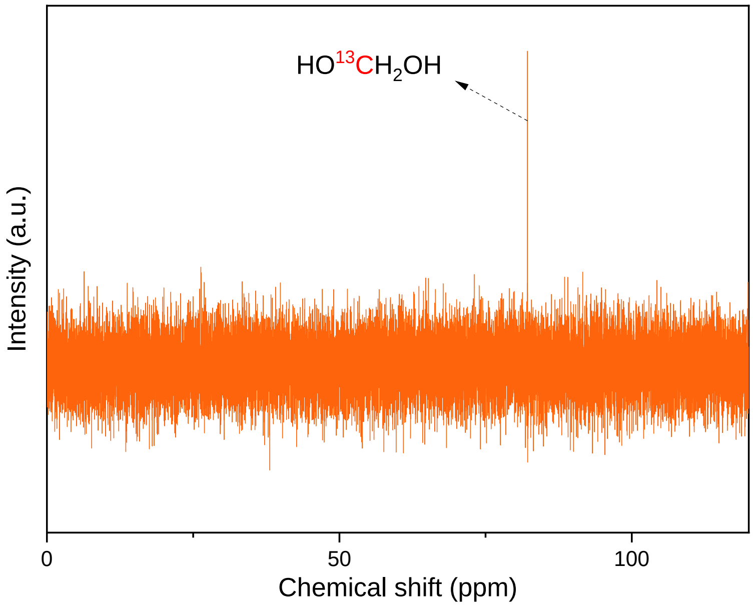

Figure S30. ^{13}C NMR spectra of the reaction product in the isotopic labeled experiment with 5 bar $^{13}CH_4$.

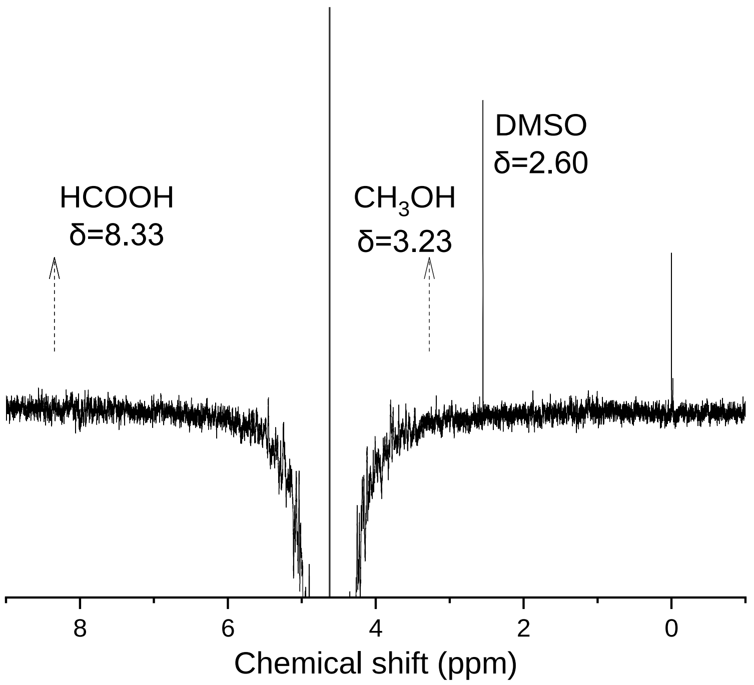

Figure S31. Representative ^{1}H NMR spectra of the products. No HCOOH and CH_3OH at δ = 8.33 and 3.23 were detected.

Table S1. EXAFS parameters of $Cu_{0.029}$-def-WO_3, CuO and Cu-foil.

Sample	Shell	Coordination number	R (Å)	$\triangle\sigma^2\times10^3$ (Å^2)	$\triangle E_0$ (eV)
Cu foil	Cu-Cu	0.9	2.54	8.68	4.6
CuO	Cu-O	3.9	1.96	3.84	1.0
	Cu-O-Cu	2.0	2.88	0.28	1.0
$Cu_{0.029}$-def-WO_3	Cu-O	3.0	1.93	4.53	-6.1

$\triangle\sigma^2$, change in the Debye-Waller factor value relative to the Debye-Waller factor of the reference compound; $\triangle E_0$*, inner potential correction to account for the difference in the inner potential between the sample and the reference compound.* $\triangle k$ *= 3.0-11.6 Å*$^{-1}$*,* $\triangle r$ *= 1.0-3.0 Å;* S_0^2 *fitting from CuO defined as* 0.85.

Table S2. Adsorption energy (in eV) of the atomic Cu on oxygen-vacancy (O_v) mediated WO_3 (002) surface, compared with that on the pristine surface.

	E_{ad} (eV)
A	-0.29
B	-0.05
C	2.15
Pristine	2.41

Table S3. Bader partial charge of W and Cu atoms in WO_3, def-WO_3 and Cu-def-WO_3.

	W	Cu	the nearest W around the O_v defect	the 2nd nearest W around the O_v defect	1st layer related O
Pristine WO_3	-1.82e	/	/	/	0.58e
Def-WO_3	/	/	-2.34e	-2.59e	1.06e
Cu-def-WO_3	/	-0.29e	-2.19e	-2.52e	0.98e

Table S4. PL lifetime of WO_3, def-WO_3 and $Cu_{0.029}$-def-WO_3.

	τ1(ns)	I1 (%)	τ2(ns)	I2 (%)	τ(ns)
WO_3	0.91	57.1	6.10	42.9	3.14
def-WO_3	0.90	54.3	6.09	45.7	3.27
$Cu_{0.029}$-def-WO_3	0.99	40.0	7.03	60.0	4.61
